# Supplementary material for: Developing and validating a cross-cultural competence scale for Japanese nurses
Source: PLoS One. 2025 Jan 9;20(1):e0310650. doi: 10.1371/journal.pone.0310650 (PMC11717215; doi:10.1371/journal.pone.0310650)
Supplement: Attachment — Submitted filename: PONE-D-23-34062_R1_reviewer.pdf [file pone.0310650.s002.pdf]

PLOS ONE

Developing and validating a cross-cultural competence scale for Japanese nurses

--Manuscript Draft--

**Manuscript Number:**	PONE-D-23-34062R1
**Article Type:**	Research Article
**Full Title:**	Developing and validating a cross-cultural competence scale for Japanese nurses
**Short Title:**	Cross-cultural competence scale for Japanese nurses
**Corresponding Author:**	Masako Sakamoto<br>Nagoya University Graduate School of Medicine Faculty of Medicine: Nagoya Daigaku Daigakuin Igakukei Kenkyuka Igakubu<br>Nagoya, Aichi JAPAN
**Keywords:**	cross-cultural competence, Japanese nurses, foreign patients, exploratory factor analysis, scale
**Abstract:**	In recent years, Japan has experienced a significant increase in the number of foreign students and workers entering the country. This has resulted in a vast number of international patients in medical facilities. This shift emphasizes the immediate need for Japanese nurses who are both clinically proficient and culturally attuned. In response, our research developed and validated the Cross-cultural Competence Scale for Japanese Nurses (CCCSJN) to better equip nurses for diverse patient care. We conducted a cross-sectional study in Japan's general hospitals using anonymous questionnaires with nurses and midwives. The scale, developed from data of 394 nurses, underwent both qualitative and quantitative evaluations to define its construct. We analyzed the data using exploratory factor analysis, criterion-related validity, internal consistency, and test-retest reliability, confirming the scale's reliability and validity. The exploratory analysis revealed five factors: "cross-cultural understanding," "cross-cultural communication skills," "motivation for cross-cultural nursing, "collaboration with multiple professions," and "respect for foreign patients." These factors explained 51.95% of the total variance. Cronbach's α for the CCCSJN was 0.92, and the test-retest reliability correlation was 0.77. The construct validity, criterion-related validity, internal consistency, and test-retest reliability of the CCCSJN were verified. The CCCSJN can be used to assess the cross-cultural competencies of Japanese nurses and identify what skills need to be mastered, leading to improved cross-cultural competence and care.
**Order of Authors:**	Masako Sakamoto
	Shigemi Iriyama, PhD
	Yu Mon Saw, PhD
**Response to Reviewers:**	Dr. Ali Khodi gave me useful advice. I thank him. In my case, I didn't understand the magazine's submission requirements, so his advice was helpful.
**Additional Information:**	
**Question**	**Response**
**Financial Disclosure**<br><br>Enter a financial disclosure statement that describes the sources of funding for the work included in this submission. Review the submission guidelines for detailed requirements. View published research articles from *PLOS ONE* for specific examples.	The author(s) received no specific funding for this work.

Powered by Editorial Manager® and ProduXion Manager® from Aries Systems Corporation

This statement is required for submission and **will appear in the published article** if the submission is accepted. Please make sure it is accurate.<br><br>**Funded studies**<br>Enter a statement with the following details:<br>• Initials of the authors who received each award<br>• Grant numbers awarded to each author<br>• The full name of each funder<br>• URL of each funder website<br>• Did the sponsors or funders play any role in the study design, data collection and analysis, decision to publish, or preparation of the manuscript?<br><br>Did you receive funding for this work?	
**Competing Interests**<br><br>Use the instructions below to enter a competing interest statement for this submission. On behalf of all authors, disclose any competing interests that could be perceived to bias this work—acknowledging all financial support and any other relevant financial or non-financial competing interests.<br><br>This statement is required for submission and **will appear in the published article** if the submission is accepted. Please make sure it is accurate and that any funding sources listed in your Funding Information later in the submission form are also declared in your Financial Disclosure statement.<br><br>View published research articles from *PLOS ONE* for specific examples.	The authors have declared that no competing interests exist.

**NO authors have competing interests**<br><br>Enter: *The authors have declared that no competing interests exist.*<br><br>**Authors with competing interests**<br><br>Enter competing interest details beginning with this statement:<br><br>*I have read the journal's policy and the authors of this manuscript have the following competing interests: [insert competing interests here]*<br><br>* typeset	
**Ethics Statement**<br><br>Enter an ethics statement for this submission. This statement is required if the study involved:<br><br>• Human participants<br>• Human specimens or tissue<br>• Vertebrate animals or cephalopods<br>• Vertebrate embryos or tissues<br>• Field research<br><br>Write "N/A" if the submission does not require an ethics statement.<br><br>General guidance is provided below. Consult the submission guidelines for detailed instructions. **Make sure that all information entered here is included in the Methods section of the manuscript.**	N/A

**Format for specific study types**<br><br>**Human Subject Research (involving human participants and/or tissue)**<br>• Give the name of the institutional review board or ethics committee that approved the study<br>• Include the approval number and/or a statement indicating approval of this research<br>• Indicate the form of consent obtained (written/oral) or the reason that consent was not obtained (e.g. the data were analyzed anonymously)<br><br>**Animal Research (involving vertebrate animals, embryos or tissues)**<br>• Provide the name of the Institutional Animal Care and Use Committee (IACUC) or other relevant ethics board that reviewed the study protocol, and indicate whether they approved this research or granted a formal waiver of ethical approval<br>• Include an approval number if one was obtained<br>• If the study involved *non-human primates*, add *additional details* about animal welfare and steps taken to ameliorate suffering<br>• If anesthesia, euthanasia, or any kind of animal sacrifice is part of the study, include briefly which substances and/or methods were applied<br><br>**Field Research**<br><br>Include the following details if this study involves the collection of plant, animal, or other materials from a natural setting:<br>• Field permit number<br>• Name of the institution or relevant body that granted permission	
**Data Availability**<br><br>Authors are required to make all data underlying the findings described fully available, without restriction, and from the time of publication. PLOS allows rare exceptions to address legal and ethical concerns. See the PLOS Data Policy and FAQ for detailed information.	Yes - all data are fully available without restriction

A Data Availability Statement describing where the data can be found is required at submission. Your answers to this question constitute the Data Availability Statement and **will be published in the article**, if accepted.<br><br>**Important:** Stating 'data available on request from the author' is not sufficient. If your data are only available upon request, select 'No' for the first question and explain your exceptional situation in the text box.<br><br>Do the authors confirm that all data underlying the findings described in their manuscript are fully available without restriction?	
**Describe where the data may be found in full sentences. If you are copying our sample text, replace any instances of XXX with the appropriate details.**<br><br>• If the data are **held or will be held in a public repository**, include URLs, accession numbers or DOIs. If this information will only be available after acceptance, indicate this by ticking the box below. For example: *All XXX files are available from the XXX database (accession number(s) XXX, XXX.).*<br>• If the data are all contained **within the manuscript and/or Supporting Information files**, enter the following: *All relevant data are within the manuscript and its Supporting Information files.*<br>• If neither of these applies but you are able to provide **details of access elsewhere**, with or without limitations, please do so. For example:<br><br>*Data cannot be shared publicly because of [XXX]. Data are available from the XXX Institutional Data Access / Ethics Committee (contact via XXX) for researchers who meet the criteria for access to confidential data.*<br><br>*The data underlying the results presented in the study are available from (include the name of the third party*	The dataset, which is the minimal basis for the study, was uploaded as a public repository by the Nagoya University Repository.<br>The uploaded data set is as follows.<br>Table 1 https://doi.org/10.18999/2008709<br>Table 2 https://doi.org/10.18999/0002008753<br>Table 3 https://doi.org/10.18999/2008754<br>Table 4 https://doi.org/10.18999/2008755<br>Table 5 https://doi.org/10.18999/2008756<br>Table 6 https://doi.org/10.18999/2008757

and contact information or URL).<br>• This text is appropriate if the data are owned by a third party and authors do not have permission to share the data.<br><br>* typeset	
Additional data availability information:	

Powered by Editorial Manager® and ProduXion Manager® from Aries Systems Corporation

Manuscript

Click here to access/download;Manuscript;Manuscriot .docx

Developing and validating a cross-cultural competence scale for Japanese nurses

Masako Sakamoto[1], Shigemi Iriyama [1], Yu Mon Saw [2]

[1]Department of Integrated Health Sciences, Nagoya University Graduate school of Medicine, Nagoya, Japan,

[2] Department of Community and Global Health, University of Tokyo, Tokyo, Japan

ORCID ID: 0009-0002-0712-8798

E-mail: sakamoto.masako.w3@s.mail.nagoya-u.ac.jp (MS)

Short title: A cross-cultural competence scale for Japanese nurses

Abstract

In recent years, Japan has experienced a significant increase in the number of foreign students and workers entering the country. This has resulted in a vast number of international patients in medical facilities. This shift emphasizes the immediate need for Japanese nurses who are both clinically proficient and culturally attuned. In response, our research developed and validated the Cross-cultural Competence Scale for Japanese Nurses (CCCSJN) to better equip nurses for diverse patient care. We conducted a cross-sectional study in Japan's general hospitals using anonymous questionnaires with nurses and midwives. The scale, developed from data of 394 nurses, underwent both qualitative and quantitative evaluations to define its construct. We analyzed the data using exploratory factor analysis, criterion-related validity, internal consistency, and test-retest reliability, confirming the scale's reliability and validity. The exploratory analysis revealed five factors: "cross-cultural understanding," "cross-cultural communication skills," "motivation for cross-cultural nursing, "collaboration with multiple professions," and "respect for foreign patients." These factors explained 51.95% of the total variance. Cronbach's α for the CCCSJN was 0.92, and the test-retest reliability correlation was 0.77. The construct validity, criterion-related validity, internal consistency, and test-retest reliability of the CCCSJN were verified. The CCCSJN can be used to assess the cross-

cultural competencies of Japanese nurses and identify what skills need to be mastered, leading to improved cross-cultural competence and care.

Introduction

There has been a noticeable upward trend in the influx of foreign workers and students into Japan in recent years, resulting in an increase in the number of foreign patients seeking care in medical facilities. According to a Ministry of Health, Labour and Welfare report, the number of foreign patients per medical facility more than doubled between 2018 and 2020, from 24.3 to 50.2 [1]. In 2021, the number of medical facilities receiving foreign patients reached a record high, with 50.1% of all facilities responsible for accommodating this influx [1].

Despite the impact of the COVID-19 pandemic, the number of foreign residents has not decreased significantly, remaining at approximately 2.82 million in 2021 [2] or 2.5% of the overall population. Since April 29, 2023, restrictions on foreign entry into Japan have eased, international travel has increased, and the number of foreign patients visiting Japanese medical facilities is expected to increase. Medical facilities that previously had minimal or no contact with foreign patients must promptly establish a

system to accommodate them [3]. Similarly, there is an increasing demand for Japanese nurses who can effectively care for foreign patients.

Culturally sensitive care must be provided to effectively address the demands of foreign patients from various cultural backgrounds [4,5]. Although 72.5% of Japanese nurses have experience in caring for foreign patients [6], these nurses acknowledged that they have not been able to provide adequate care. Japanese nurses often experience anxiety and difficulty in language and communication [7–10], as well as difficulties in addressing cultural aspects such as lifestyle, diet, and religious practices [11,12].

To ensure the safe and uncomplicated delivery of medical care to foreign patients, nurses' competencies in interacting with culturally diverse individuals is critical. This goes beyond fundamental communicative skills, necessitating a sophisticated comprehension of various cultural factors such as lifestyle choices, dietary preferences, and religious practices [13]. Unfortunately, Japanese nurses are often hesitant to provide care to foreign patients because of the bewilderment caused by differences in communication, culture, and customs. A lack of cross-cultural competence can affect the quality of nursing care [14] and reduce patient satisfaction and safety [15]. However, if nurses can properly assess their own cross-cultural competence, they can

visualize what competencies are lacking. This increased self-awareness can improve the quality of care they provide to foreign patients.

Globally, numerous studies on nursing care for foreign patients have produced various scales to measure nurses' cross-cultural competence. Widely used scale concepts include three-dimensional models, such as the scale of beliefs and attitudes, knowledge, and skills developed by Suh et al. in the United States [13]. While several measures of cross-cultural competence have been properly tested and shown to be reliable, many lack the necessary validation [16, 17]. It is essential to employ appropriately validated scales to assess nurses' cross-cultural competencies.

Currently, only a few scales assess the cross-cultural competence of Japanese nurses. These include Cultural Competence in Nursing (CCN) [18] and the Japanese version of the Caffrey Cultural Competence Health Services (J-CCCHS) [6]. The CCN was first developed in Japan by Sugiura in 2003, referring to the Campinha–Bacote conceptual model [19,20]. Although construct validity was shown, the Cronbach's α for one factor was low (0.64). Additionally, the number of questions was large (n = 46), raising concerns about the burden on participants. Therefore, the validity and reliability of the scale needs further examination. Further, the J-CCCHS was developed through a large-scale survey conducted by Noji in 2017. Structural equation modeling was used for

the analysis, but the scale is not yet practical owing to insufficient model fit [6]. Further improvements are urgently needed to create a valid and reliable measure for Japanese nurses.

The study purpose was therefore to develop and validate the Cross-cultural Competence Scale for Japanese Nurses (CCCSJN).

Materials and methods

Preliminary study

To develop the CCCSJN, it was necessary to use questionnaire items that reflect the characteristics of Japanese nurses. Therefore, a preliminary qualitative study was conducted. The results were then used as items in the questionnaire.

We conducted 30-minute semi-structured interviews based on interviews with 14 Japanese nurses with experience in caring for foreign patients after obtaining informed consent. We analyzed the concept of cross-cultural responsiveness from data recorded by IC recorders and extracted eight categories: consideration for foreign patients, motivation to care for foreign patients, ingenuity in explanation, utilization of social resources, confirmation, dignity, financial support, and collaboration. We then drafted the 40-item CCCSJN.

To assess content validity, four university faculty members working as cross-cultural nursing researchers examined the clarity of each item and its relevance to the concept. Three items were removed because of duplication and ambiguity, leaving a final draft of 37 items in the aforementioned eight categories. The overall content validity index of the scale was 0.96.

Study design and study duration

We conducted a cross-sectional study using an anonymous self-administered questionnaire between August 2022 and January 2023.

Study participants

We examined nurses and midwives in general hospitals located in four cities in Aichi Prefecture with many foreign residents. The selection criteria for participants were nurses and midwives of Japanese nationality. Outpatient and ward nurses were excluded because administrative responsibilities greatly expanded their roles beyond simply providing care.

Sampling procedures and data collection

Japanese nurses were recruited through convenience sampling from six medical facilities with more than 200 beds. We selected Aichi Prefecture as the site for this study because it has the second-largest number of foreign residents after Tokyo. Consequently,

nurses working in Aichi Prefecture were speculated to have more experience caring for foreign patients than those in other prefectures. In total, there were 165 participating medical facilities. However, the number of eligible medical facilities was 27, excluding psychiatric, oncology, pediatric, and recuperation facilities and medical facilities with less than 200 beds. Research cooperation was requested in writing from the nursing directors of these 27 medical facilities, and consent was obtained from the nursing directors of six medical facilities. Nursing directors in the outpatient and ward departments distributed study request forms, two anonymous self-report questionnaires, and post-paid envelopes to the participants. We collected data by mail.

Definition of "cross-cultural competence"

"Cross-cultural competence" was operationally defined based on the three-dimensional model proposed by Suh et al. [13]. This term indicates the ability to provide appropriate nursing care for foreign patients from different cultural backgrounds. This ability requires cross-cultural knowledge, communication skills, and responding to foreign patients with respect.

Study measures

Social critical thinking orientation (SCTO)

To verify the criterion-related validity of the CCCSJN, we used two SCTO subscales—"understanding diversity" and "understanding others"—developed by Nakanishi et al. [21]. The SCTO comprises 27 items and seven factors: emphasis on logic and evidence, discrediting, understanding others, understanding diversity, authenticity, understanding key points, and determination. Permission to use the SCTO was obtained from the authors via email.

The subscales "understanding diversity" and "understanding others" correspond to the "power to understand people" and the "power to practice people-centered care" among the elements of nursing practical ability [22]. Therefore, the two subscales were adopted to infer that they were related to the cross-cultural ability of Japanese nurses to provide the necessary care to foreign patients. Both "understanding diversity" and "understanding others" comprise four items and are rated on a seven-point Likert scale from "not at all applicable" (1) to "very applicable" (7). We calculated the sum score, with higher scores indicating a higher degree of understanding diversity and others.

Japanese intercultural sensitivity scale (J-ISS)

To verify the criterion-related validity of the CCCSJN, we used a subscale of the J-ISS [23]: "positive feelings for different cultures." The ISS was developed by Chen and Starosta [24], and the J-ISS was developed by Suzuki et al. [20]. The J-ISS comprises 22

items and three concepts: “positive feelings toward different cultures,” “ambivalent feelings toward different cultures,” and “negative feelings toward different cultures.” Permission to use the J-ISS was obtained from the authors via email.

“Positive feelings toward different cultures” comprises items with positive emotional content, such as actively wanting to know about cultural differences or enjoying cultural differences [23]. We speculated that this concept is related to cross-cultural competence needed by Japanese nurses caring for foreign patients and was adopted as the criterion-related validity of the CCCSJN. The J-ISS subscale “positive feelings toward different cultures” comprises 10 items with a five-point Likert scale ranging from “not at all” (1) to “very true” (5). We calculated the sum of the scores, with high scores indicating a high degree of positivity.

Socio-demographic characteristics

We collected socio-demographic information such as age, years of work experience, department affiliation, nursing qualifications, and educational background.

Cross-cultural experience in nursing

We collected data on the frequency of foreign patient care, experience of living abroad for more than a month, contact with foreigners, foreign language proficiency, and experience in cross-cultural education.

Statistical analysis

Age and years of nursing experience were not normally distributed and thus were calculated with median and interquartile range (IQR).

The construct validity of the CCCSJN was assessed using exploratory factor analysis. An item analysis was performed beforehand to examine ceiling and floor effects and item-total correlations. The principal factor method was selected for factor extraction and promax rotation was used because there was a correlation between the subscales. The statistical criteria determined the extraction of factors with eigenvalues of ≥ 1. Based on these analyses, items with loadings ≥ 0.40 were considered significant and factors were extracted.

To verify the criterion-related validity of the CCCSJN, the relationships between the subscales of the CCCSJN and the SCTO ("understanding others" and "understanding diversity") and between the subscales of the CCCSJN and the J-ISS ("positive feelings toward different cultures") were examined using Pearson's correlation coefficients.

The reliability (internal consistency) of the CCCSJN was determined by Cronbach's alpha coefficients. The test-retest reliability of the first and second surveys was indicated by intraclass correlation coefficients (ICCs).

Data were analyzed using SPSS version 22.0 (IBM SPSS Inc.).

Ethical considerations

We obtained ethical approval from the ethics committee of Nagoya University School of Medicine (no. 22-108) and the six medical facilities. The study purpose was fully explained to all participants, and participants were not obliged to participate in this study and could withdraw at any time. In order to consent to the research, a consent box was provided at the beginning of the questionnaire, and participants were asked to check the box if they consented.

Results

Descriptive statistics

In the first study, 406 out of 504 (72.2% effective response rate) responded by mail, and 394 out of 406 (97.0% effective response rate) responded to the CCCSJN questionnaire. All participants were women. The median age of the nurses was 33.0 years (IQR: 26–44), and their nursing experience was 9.8 years (IQR: 4.6–19.7). Of these, 330 (83.8%) completed their academic career at vocational schools and 51 (13.0%) had graduated from universities or graduate schools. There were 317 (80.5%) nurses working at city hospitals and 19 (4.8%) working at university hospitals (Table 1).

Table1 Japanese nurses' socio-demographic characteristics (N=394)

		n	%	Median	(IQR)
Age（years）				33	(26-44)
Length of clinical experience (years)				9.8	(4.6-19.7)
Qualification					
	Nurse	372	94.4		
	Midwife	22	5.6		
Educational background					
	Diploma	330	83.7		
	Bachelor's degree	44	11.2		
	Advanced diploma	9	2.3		
	Master's degree	7	1.8		
	Associate degree	4	1.0		
Department					
	Inpatient	321	81.5		
	Outpatient	73	18.5		
Number of nurses by employment facility					
	Municipal hospital	317	80.5		
	Consumers' co-operative hospital	46	11.7		
	University hospital	19	4.8		
	Public interest incorporated foundation hospital	12	3.0		

IQR=Interquartile range

https://doi.org/10.18999/2008709

There were 12 (3.0%) nurses who had at least one month's experience of Living overseas, while five (1.3%) had overseas training or work experience. Fifty-four (13.7%) nurses had contact with foreigners other than patients in their daily lives, and 22 (5.6%) nurses had foreign skills equivalent to daily conversations. Ninety-five (24.2%) nurses had cross-cultural understanding and learning experiences, while 325 nurses (82.5%) had cared for one or more foreign patients in the past year. The median number of foreign patients in the past year were five (IQR: 3–10; Table 2).

Table 2 Japanese nurses' cross-cultural experience (N=394)

		n	%	Median	(IQR)
Stay abroad [a] (≧1month)	Yes	12	3.1		
	No	380	96.9		
Abroad nursing experience (Training/Work)	Yes	5	1.3		
	No	389	98.7		
Contact with foreigners other than foreign patients	Yes	54	13.7		
	No	340	86.3		
Conversational proficiency in a foreign language	Yes	22	5.6		
	No	372	94.4		
Cross-cultural learning [b]	Yes	95	24.2		
	No	298	75.8		
Experience of foreign patient care in the year	Yes	325	82.5		
	No	69	17.5		
Experience of foreign patient care in the year [c]				5	(3-10)

IQR= Interquartile range [a] Missing value：2 [b] Missing value：1 [c] Missing value：76

https://doi.org/10.18999/0002008753

Validity and reliability of the CCCSJN

CCCSJN construct validity

To confirm the construct validity of the CCCSJN, item analysis was performed before the exploratory factor analysis. The distribution showed a ceiling effect for Q19 (5.09), Q20 (5.01) and Q21 (5.08), while Q4 showed a floor effect (0.75). The I-T correlation was less than 0.30 for Q4 (0.06), Q9 (0.23), Q20 (0.29), and Q21 (0.21). Therefore, five of the 37 items were excluded from the CCCSJN(Table3).

Table 3 Item Analysis of the CCCSJN for Japanese nurses (N=394)

	Question items	Mean	SD	CE	FE	I-T
Q1	I treat foreign patients with respect.	4.02	0.72	4.74	3.30	0.56
Q2	I frequently talk to foreign patients.	3.40	0.90	4.30	2.50	0.60
Q3	I greet foreign patients in their native languages as much as possible.	2.68	1.11	3.79	1.57	0.38
Q4	When foreign patients are admitted to a ward, I always introduce them to the patients in the same room.	1.69	0.94	2.63	0.75	0.06
Q5	I try not to show foreign patients that I am in a hurry.	3.15	0.86	4.01	2.30	0.34
Q6	When I cannot satisfy the wishes of a foreign patient, I explain the reason and obtain his/her understanding.	3.54	0.84	4.38	2.70	0.53
Q7	When a foreign patient asks me a question about nursing care, I provide a detailed explanation.	3.61	0.80	4.42	2.81	0.58
Q8	I listen to foreign patients carefully and try to understand their thoughts.	3.86	0.70	4.57	3.16	0.66
Q9	When I know I'll be dealing with foreign patients in advance, I try to research the culture of the country they come from.	2.22	1.03	3.25	1.19	0.23
Q10	I try to provide the same care to foreign patients as I do to Japanese patients.	4.11	0.77	4.88	3.34	0.51
Q11	I apply the experiences I have learned from foreign patients in my nursing care.	3.16	0.86	4.02	2.31	0.52
Q12	When there is a difference between the wishes of foreign patients and the rules of the hospital, I think of other ways to address the issue together with the patient.	2.87	0.92	3.78	1.95	0.47
Q13	I want to be actively involved in the care of foreign patients.	2.87	0.96	3.83	1.91	0.49
Q14	I take time to explain matters to foreign patients.	3.45	0.85	4.30	2.60	0.63
Q15	I speak slowly to foreign patients in simple Japanese.	4.13	0.65	4.77	3.49	0.45
Q16	I use gestures to explain things to foreign patients who do not understand Japanese.	4.27	0.67	4.94	3.60	0.52
Q17	I explain to foreign patients by showing them illustrations and drawings.	3.44	1.01	4.45	2.43	0.35
Q18	I use a comparison chart for foreign patients in their native language or in an understandable foreign language and Japanese.	3.49	1.12	4.61	2.37	0.36
Q19	I use translation devices and translation apps for foreign patients.	4.10	0.99	5.09	3.11	0.34
Q20	I get the cooperation of family and friends who understand Japanese.	3.99	1.02	5.01	2.97	0.29
Q21	I always use medical interpreters when I give IC or important explanations to foreign patients.	3.99	1.09	5.08	2.90	0.21
Q22	I check the thoughts and intentions of foreign patients before providing care.	3.45	0.87	4.32	2.58	0.58
Q23	I observe the facial expressions and reactions of foreign patients to see if they understand what I want to convey.	4.06	0.63	4.68	3.44	0.66
Q24	I ask questions to make sure foreign patients understand what I have tried to convey.	3.72	0.81	4.53	2.91	0.39
Q25	I understand that the cultures of foreign patients may make them have different views regarding health and disease matters.	3.52	0.88	4.40	2.64	0.62
Q26	I understand that different cultures have different values.	3.89	0.77	4.66	3.12	0.69
Q27	I understand that foreign patients'religion or beliefs may dictate traditional diets or dietary restrictions.	4.03	0.73	4.76	3.30	0.69
Q28	I understand that foreign patients may have behavioral restrictions or rules according to their religion.	3.95	0.74	4.69	3.21	0.69
Q29	I understand that culturally sensitive nursing care must be provided to all foreign patients.	3.92	0.77	4.69	3.15	0.70
Q30	I understand that foreign patients who are here for a short period of stay may feel anxious about their hospital stay.	3.88	0.82	4.71	3.06	0.68
Q31	I understand that the health care system in foreign patients' country of origin is different.	3.50	0.94	4.44	2.56	0.61
Q32	I understand that foreign patients may have financial concerns.	3.79	0.78	4.57	3.01	0.63
Q33	I share information with the staff in my office in order to understand foreign patients. .	3.60	0.84	4.44	2.76	0.63
Q34	I have detailed information on the daily lives of foreign patients in my medical records.	3.15	0.85	4.00	2.30	0.52
Q35	I do not handle foreign patients by myself, I collaborate with my colleagues in the office.	3.79	0.75	4.54	3.04	0.62
Q36	I cooperate with staff from various professions to meet the wishes of foreign patients.	3.75	0.75	4.51	3.00	0.59
Q37	With the consent of the foreign patient, I share information with supporters.	3.55	0.81	4.36	2.74	0.54

CE=ceiling effect; FE=floor effect; I-T=i-t correlation

https://doi.org/10.18999/2008754

In the first exploratory factor analysis, Q5, Q18, Q22, and Q24 were excluded because their factor loadings were less than 0.4. The factor loading for Q8 was 0.38, but it was borderline and similar to the factor concepts in questions 6 and 7; therefore, it was not excluded and was reserved. In the second exploratory factor analysis, all questionnaire items were above 0.40. The factor loading of Q17 was 0.65, but it was excluded because it was one factor and one item (Table 4). To ensure that exclusion criterion of less than 0.40 was reasonable, an exploratory factor analysis was performed by excluding items with a factor loading of less than 0.35. The result was a six-factor structure with 29 items. However, one factor included only two items: Q17 and Q18. Cronbach's α was low as 0.53.

Table 4 Factor loadings on CCCSJN for Japanese nurses after items analysis (N=394)

32 Items	1	2	3	4	5	6
Q26. I understand that different cultures have different values	**0.86**	0.11	0.03	-0.11	-0.12	0.05
Q28. I understand that foreign patients may have behavioral restrictions or rules according to their religion.	**0.86**	-0.07	0.19	-0.11	-0.03	0.00
Q27. I understand that foreign patients' religion or beliefs may dictate traditional diets or dietary restrictions	**0.82**	-0.07	0.22	-0.11	-0.04	-0.01
Q31. I have detailed information on the daily lives of foreign patients in my medical records.	**0.76**	0.06	-0.27	0.14	0.00	-0.10
Q29. I understand that culturally sensitive nursing care must be provided to all foreign patients.	**0.75**	-0.08	0.12	0.03	0.06	-0.04
Q32. I understand that foreign patients may have financial concerns.	**0.69**	-0.07	-0.13	0.19	0.11	-0.14
Q25. I understand that the cultures of foreign patients may make them have different views regarding health and disease matters.	**0.65**	0.28	-0.20	0.00	-0.11	0.13
Q30. I understand that foreign patients who are here for a short period of stay may feel anxious about their hospital stay.	**0.64**	-0.13	0.04	0.09	0.13	0.09
Q13. I want to be actively involved in the care of foreign patients.	-0.04	**0.85**	0.04	-0.04	-0.21	0.03
Q2. I frequently talk to foreign patients.	-0.15	**0.62**	0.25	0.04	0.03	-0.15
Q3. I greet foreign ptients in their native languages as much as possible.	-0.03	**0.53**	-0.07	-0.10	0.03	0.14
Q11. I apply the experiences I have learned from foreign patients in my nursing care.	0.15	**0.49**	-0.09	-0.01	-0.03	0.10
Q12. When there is a difference between the wishes of foreign patients and the rules of the hospital, I think of other ways to come to terms with them together with foreign patients.	0.07	**0.44**	-0.20	0.06	0.15	0.14
Q14. I take time to explain to foreign patients.	0.11	**0.43**	0.17	0.00	0.10	0.01
Q22. I check the thoughts and intentions of foreign patients before providing care.	0.07	0.07	0.07	0.07	0.07	0.07
Q24. I ask questions to make sure foreign patients understand what I have tried to convey.	0.26	0.28	0.13	0.07	0.11	0.00
Q15. I speak slowly to foreign patients in simple Japanese.	-0.06	-0.10	**0.75**	0.00	0.01	0.07
Q16. I use gestures to explain things to foreign patients who do not understand Japanese.	0.12	-0.11	**0.69**	0.04	-0.09	0.19
Q1. I treat foreign patients with respect.	-0.07	0.30	**0.58**	0.11	-0.14	-0.15
Q10. I try to provide the same care to foreign patients as I do to Japanese patients.	-0.01	0.08	**0.53**	-0.02	0.12	-0.09
Q23. I observe the facial expressions and reactions of foreign patients to see if they understand what I want to convey.	0.33	-0.03	**0.41**	0.03	0.06	0.04
Q37. With the consent of the foreign patient, I share information with supporters.	0.03	-0.06	0.01	**0.78**	-0.12	0.15
Q36. I cooperate with staff from various professions to meet the wishes of foreign patients.	-0.09	-0.09	0.12	**0.71**	0.06	0.13
Q33. I share information with staff in my office in order to understand foreign patients.	0.25	0.04	-0.06	**0.68**	-0.09	-0.15
Q35. I do not handle foreign patients by myself, I collaborate with my colleagues in the office.	0.02	-0.09	0.21	**0.65**	0.00	-0.06
Q34. I have detailed information on the daily lives of foreign patients in my medical records.	-0.08	0.20	-0.15	**0.53**	0.10	0.10
Q7. When a foreign patient asks me a question about nursing care, I provide a detailed explanation.	0.03	-0.01	-0.03	-0.09	**0.92**	-0.08
Q6. When I cannot satisfy the wishes of a foreign patient, I explain the reason and obtain his/her understanding.	-0.07	-0.11	-0.01	0.05	**0.80**	0.08
Q8. I listen to foreign patients carefully and try to understand their thoughts.	0.01	0.22	0.28	-0.03	**0.38**	0.08
Q5. I try not to show foreign patients that I am in hurry.	0.02	0.22	0.00	-0.05	0.29	-0.05
Q17. I explain to foreign patients by showing them illustrations and drawings.	-0.05	0.13	0.02	0.08	-0.01	**0.65**
Q18. I use a comparison chart for foreign patients in their native language or in an understandable foreign language and Japanese.	0.00	0.12	0.12	0.11	-0.01	0.36
Eigen values	10.33	1.91	1.36	1.08	0.81	0.65
Percentage of variance	32.29	5.98	4.25	3.37	2.53	2.05
Cumulative of total variance explained	32.29	38.27	42.52	45.89	48.42	50.46

https://doi.org/10.18999/2008755

A third exploratory factor analysis was performed, and the criterion was less than 0.40. Finally, a five-factor structure comprising 27 items was adopted. The model fit showed that the validity of the Kaiser–Meyer–Olkin sample was 0.918, and Bartlett's sphericity test showed a good fit ($p < 0.001$). The cumulative contribution rate was 51.95 (Table 5).

Table 5 Factor loadings on CCCSJN for Japanese Nurses (N=394)

**27 items α=0.92**	**1**	**2**	**3**	**4**	**5**
**Foctor1：Understanding of cross- cultural (α=0.91)**					
Q28. I understand that foreign patients may have behavioral restrictions or rules according to their religion.	**0.85**	0.20	-0.07	-0.11	-0.04
Q26. I understand that different cultures have different values	**0.85**	0.04	0.10	-0.08	-0.11
Q27. I understand that foreign patients'religion or beliefs may dictate traditional diets or dietary restristions	**0.82**	0.23	-0.06	-0.12	-0.05
Q31. I have detailed information on the daily lives of foreign patients in my medical records.	**0.74**	0.13	-0.07	0.00	0.05
Q29. I understand that culturally sensitive nursing care must be provided to all foreign patients.	**0.73**	-0.28	0.08	0.11	0.01
Q32. I understand that foreign patients may have financial concerns.	**0.67**	-0.11	-0.03	0.12	0.11
Q25. I understand that cultures of foreign patients may have different views regarding health and disease matters.	**0.65**	-0.19	0.26	0.04	-0.08
Q30. I understand that foreign patients with a short period of stay may feel anxous about their hospital stay.	**0.63**	0.04	-0.11	0.11	0.14
**Factor2：Cross-cultural communication ability (α=0.78)**					
Q15. I speak slowly to foreign patients in simple Japanese.	-0.06	**0.76**	-0.10	0.03	0.00
Q16. I use gestures to explain things to foreign patients who do not understand Japanese.	0.12	**0.67**	-0.11	0.10	-0.05
Q1. I treat foreign patients with respect.	-0.07	**0.58**	0.30	0.04	-0.12
Q10. I try to provide the same care to foreign patients as I do to Japanese patients.	-0.01	**0.53**	0.09	-0.05	0.12
Q23. I observe the facial expressions and reactions of foreign patients to see if they understand what I want to convey.	0.33	**0.41**	-0.05	0.06	0.04
**Factor3：Motivation for cross-cultural nursing (α=0.75)**					
Q13. I want to be actively involved in the care of foreign patients.	-0.02	0.07	**0.82**	-0.03	-0.17
Q2. I frequently talk to foreign patients.	-0.13	0.27	**0.59**	-0.01	0.04
Q3. I greet foreign patients in their native languages as much as possible.	-0.01	-0.06	**0.47**	-0.02	0.04
Q11. I apply the experiences I have learned from foreign patients in my nursing care.	0.17	-0.08	**0.47**	0.03	0.00
Q12. When there is a difference between the wishes of foreign patients and the rules of the hospital, I think of other ways to address the issue together with the patient.	0.10	-0.18	**0.42**	0.10	0.15
Q14. I take time to explain matters to foreign patients.	0.12	0.19	**0.41**	0.01	0.12
**Factor4：Cooperation with multiple professions (α=0.82)**					
Q37. With the consent of the foreign patient, I share information with supporters.	0.02	0.02	-0.04	**0.81**	-0.10
Q36. I cooperate with staff from various professions to meet the wishes of foreign patients.	-0.08	0.14	-0.06	**0.71**	0.06
Q35. I do not handle foreign patients by myself alone, but collaborate with my colleagues in the office.	0.01	0.23	-0.04	**0.60**	-0.01
Q33. I share information closely with staff in my office in order to understand foreign patients. .	0.23	-0.04	0.07	**0.58**	-0.08
Q34. I have detailed information on the daily lives of foreign patients in my medical records.	-0.06	-0.13	0.20	**0.54**	0.11
**Factor5：Respect for foreign patiens (α=0.79)**					
Q7. When a foreign patient asks me a question about nursing care, I explain the reason in detail.	0.03	-0.03	0.02	-0.11	**0.93**
Q6. When I cannot satisfy the wishes of a foreign patient, I explain the reason and obtain his/her understanding.	-0.05	0.03	-0.07	0.11	**0.69**
Q8. I listen to foreign patients carefully and try to understand their thoughts.	0.03	0.27	0.22	-0.01	**0.42**
Eigen values	9.17	1.76	1.34	0.99	0.76
Percentage of variance	33.98	6.53	4.95	3.66	2.83
Cumulative of total variance axplained	33.98	40.51	45.46	49.12	51.95

https://doi.org/10.18999/2008756

The first factor contributed 33.98%, the second factor 6.53%, the third factor 4.95%, the fourth factor 3.66%, and the fifth factor 2.83%. The factor loadings were

0.63–0.85 for eight items on the first factor, 0.41–0.76 for five items on the second factor, 0.41–0.82 for six items on the third factor, 0.55–0.81 for five items on the fourth factor, and 0.42–0.93 for three items on the fifth factor. We named the first factor “cross-cultural understanding,” the second factor “cross-cultural communication skills,” the third factor “motivation for cross-cultural nursing,” the fourth factor “collaboration with multiple professions,” and the fifth factor “respect for foreign patients.”

Criterion-related validity of the CCCSJN

The total CCCSJN score showed a significant positive correlation with the SCTO subscales “understanding diversity” and “understanding others” (understanding diversity: $r = 0.51$, $p < .001$; understanding others: $r = 0.47$, $p < .001$). The total score of CCCSJN also showed a significant positive correlation with the J-CCSS subscale “positive feelings toward different cultures” ($r = 0.61$, $p < .001$; Table 6).

Table 6 Correlation between total scores of the CCCSJN and two subscales of the SCTO, a subscale of J-ISS

N=390

	CCCSJN	
	r	p
1. The Social Critical Thinking Orientation: subscale **diversity understanding**	0.51	$<.001$
2. The Social Critical Thinking Orientation: subscale **other understanding**	0.47	$<.001$
3. Japanese version of the Intercultural Sensitivity Scale: subscale **positive feelings toward different cultures**	0.61	$<.001$

r = Pearson correlation coefficient p = probability value

https://doi.org/10.18999/2008757

Internal consistency of CCCSJN

The Cronbach’s α for the overall CCCSJN was 0.92 (Table 5). Cronbach's α for each subscale of CCCSJN were 0.91 for “cross-cultural understanding,” 0.78 for “cross-cultural communication skills,” 0.75 for “motivation for cross-cultural nursing,” 0.82 for “collaboration with multiple professions,” and 0.79 for “respect for foreign patients.” There was a strong correlation between the CCCSJN total score and all subscale factors ($rs = 0.70–0.84$, $p < .001$). Moderate correlations existed between each subscale ($rs = 0.44–0.56$, $p < .001$).

CCCSJN test-retest reliability

Of the 394 participants, 341 returned the questionnaire in the second study (response rate = 60.8%). Substantial correlations were found between the first and second studies (ICC [1,1] = 0.767 [0.72–0.81], $p < .001$).

Discussion

This study developed a scale—the CCCSJN—to measure the cross-cultural capacity of Japanese nurses to care for foreign patients. The scale demonstrated construct validity, criterion-related validity, internal consistency, and test-retest reliability. The CCCSJN had a five-factor structure, in which the third, fourth, and fifth factors reflected

the cultural characteristics of Japanese nurses. The third factor, “motivation for cross-cultural nursing,” was the concept of nurses learning about cultural diversity and being positive about actively engaging with patients from different cultural backgrounds. In a previous study [25], a significant positive association was found between work attitude and motivation. Thus, it can be inferred that the concept of “motivation for cross-cultural nursing” promotes the ability of Japanese nurses to cope with cross-cultural situations. In Germany, a scale expressed as the concept of motivation and curiosity was developed [26], validated, and was deemed sufficiently reliable for adoption as a high-impact primary factor. This concept is also similar to the “cultural desires” of Campinha–Bacote’s conceptual model. However, only a few scales adopted the concept of “cultural desires” [27,28], and the validity and reliability were poorly tested.

The fourth factor, “collaboration with multiple professions,” was not represented by existing scales in Europe, the United States, and Japan, confirming that this concept is unique to the CCCSJN. This means that nurses do not have to manage foreign patients on their own but can share information closely with staff at their workplace and collaborate with a wide range of professionals to ensure that they respond to the needs of foreign patients. Compared to the West, Japanese culture favors a mutually cooperative self-view [29,30], valuing cooperative relationships

between individuals [31]. Japanese people are also more interested in group goals and cooperative behavior. Although “collaboration with multiple professions” is essential for nurses, it was particularly evident in the CCCSJN.

The fifth factor, “respect for foreign patients,” refers to the attitude of responding to foreign patients with respect for their wishes and intentions. The Japanese are often characterized as having a national character that emphasizes respect for others, values compassion and courtesy, and may prioritize the needs of others over their own. In a 2019 Ministry of Land, Infrastructure, Transport, and Tourism attitude survey, more than 80% of respondents said it was important to be respectful and considerate of others [32]. “Respect for foreign patients” was an important attitude for nurses and a concept that emerged prominently in the CCCSJN.

“Use of social resources” was not included in the five-factor CCCSJN, although it was suggested that many Japanese nurses use social resources. “Use of a translation device,” “cooperation with family and friends of patients who could understand Japanese,” and “use of medical interpreters” were excluded at an early stage because of the ceiling effect. This meant that many Japanese nurses scored the highest.

Proof of criterion-related validity suggests that the CCCSJN could be an

appropriate measure of cross-cultural competence. Total CCCSJN score was significantly positively correlated with the SCTO subscales “understanding diversity” and “understanding others.” These questions were similar to those in the first factor of the CCCSJN, “cross-cultural understanding,” and were useful for understanding foreign patients. Japanese nurses also tended to emphasize “understanding diversity” and “understanding others” even in their nursing practice for Japanese patients [22]. The total CCCSJN score also showed a significant positive correlation with the J-CCSS subscale “positive feelings toward different cultures,” which was related to flexibility and adaptability in cross-cultural situations, and meaningful communication inspired by responses from others [23].

The CCCSJN had high internal consistency. Specifically, the Cronbach’s α for all CCCSJN items was high at 0.92, and the five subscales also showed high internal consistency at 0.75–0.91, indicating high reliability. The other scales showed high internal consistency, similar to that of the CCCSJN. The J-CCCHS showed Cronbach’s α for the five subscales ranged from 0.76–0.90 [6]. The Nursing Cultural Competence Scale had Cronbach’s α for the four-subscale that ranged from 0.82–0.84 [33]. Moderate to strong correlations between the CCCSJN and subscales was also obtained ($rs = 0.68–0.84$, $p < .001$). Additionally, a strong positive correlation was found

between the first and second studies on the test-retest reliability of the CCCSJN, indicating the stability of the scale.

Study limitations

This study has three limitations. The first limitation is selection bias because we recruited at medical facilities in which foreign patients are common. A multistage sampling study of 5430 nurses nationwide in Japan found that 72.5% had experienced caring for foreign patients [6], compared with 82.5% in this study. It is difficult to generalize the current findings to all Japanese nurses because this may not apply to those working in facilities in areas where there are few foreign residents. However, the current results could be applicable to nurses in urban areas, such as the Kanto and Kansai regions.

Second, recall bias could have occurred because some questions required recalling the content of care for foreign patients and the number of times they administered such care in a year. The recall period must be considered to avoid memory ambiguity and ensure accurate answers.

Third, female nurses were the only participants. The percentage of male nurses in Japan is 8.1%, which is about two times higher than 10 years ago [34], and this trend is expected to continue. Future studies including male nurses are warranted.

Conclusions

This study was the first to use a semi-structured interview method to develop a scale of cross-cultural competence for Japanese nurses caring for foreign patients. The scale has a five-factor structure and demonstrated construct validity, criterion-related validity, internal consistency, and test-retest reliability. Therefore, the scale is useful for assessing cross-cultural competence. The CCCSJN can be employed to assess the cross-cultural competencies of Japanese nurses and identify those that need to be mastered, leading to improved cross-cultural competencies and care.

Acknowledgments

We express our sincere appreciation to all the Japanese nurse from the six medical facilities in Aichi, Japan for their kind support and active cooperation in this study.

Authors' contributions

Conceptualization: Masako Sakamoto, Shigemi Iriyama, Yu Mon Saw

Data Curation: Masako Sakamoto, Shigemi Iriyama

Formal Analysis: Masako Sakamoto, Shigemi Iriyama, Yu Mon Saw

Funding acquisition: Masako Sakamoto

Investigation: Masako Sakamoto, Shigemi Iriyama, Yu Mon Saw

Methodology: Masako Sakamoto, Shigemi Iriyama, Yu Mon Saw

Project Administration: Masako Sakamoto, Shigemi Iriyama

Resources: Masako Sakamoto

Supervision: Masako Sakamoto, Shigemi Iriyama, Yu Mon Saw

Validation: Masako Sakamoto, Shigemi Iriyama

Visualization: Masako Sakamoto, Shigemi Iriyama

Writing – original draft: Masako Sakamoto, Shigemi Iriyama, Yu Mon Saw

Writing – review & editing: Masako Sakamoto, Shigemi Iriyama, Yu Mon Saw

References

1. Ministry of Health, Labour and Welfare. Reiwa actual situation regarding the acceptance of foreign patients at medical institutions in the third year. https://www.mhlw.go.jp/stf/newpage_25805.html.

2. Immigration Services Agency. Number of foreign residents as of June 2021. https://www.moj.go.jp/isa/publications/press/1300017.html

3. Ministry of Health, Labour and Welfare. Fact-finding Survey on the Acceptance of f foreign patients at Medical Institutions Result Report. https://www.mhlw.go.jp/content/10800000/000940326.pdf.

4. International Association of Nurses (ICN). Code of Ethics for ICN Nurses (2021 Edition). https://www.nurseor.jp/nursing/international/icn/document/ethics /index.html.

5. Japan Nursing Association: Code of Ethics for Nurses. https://www.nurse.or.jp/nursing/practice/rinri/rinri.html.

6. Noji A, Mochizuki Y, Nosaki A, Glaser D, Gonzales L, Mizobe A, et al. Evaluating cultural competence among Japanese clinical nurses: Analyses of a translated scale. Int J Nurs Pract. 2017;23: Suppl 1. https://doi.org/10.1111/ijn.12551

7. Inoue C, Matsui M, Lee S, Nakamura N, Minoura S, Ushijima H. Research into the problems faced by and support given to non-Japanese speakers in need of perinatal care: From an analysis of the deliveries in one hospital over a 12-year period. J Int Health. 2006;21(1): 25-32. https://www.researchgate.net/publication/344014266

8. Iwata M, Yuki H. A literature review of the challenges and measures to promote cross-cultural communication in nursing practice. Journal of Japanese Society for International Nursing. 2021;(2): 12-22. https://www.jsin.jp/PDF/2021-2_paperNo2ENG. Pdf

9. Maeno M, Sakuyama M, Motoyama S, Matsuo H. Japanese nurses' view of perioperative management of foreign patients in Osaka. J Int Health. 2011;26(4): 272-280. https://doi.org/10.11197/JAIH.26.273

10. Nonaka C, Higuchi M. The process of building relationships between foreign patients and nurses in Japan. J Int Health. 2010;25(1): 21-32. https://doi.org/10.11197/jaih.25.21.

11. Teraoka M, Muranaka Y. Aspects of cross-cultural experience perceived by foreigners living in Japan when using its healthcare services. J Jpn Acad Nurs Sci. 2017;37: 35-44. https://doi.org/10.5630/jans.37.35.

12. Itoi Y. Feeling with the use of medical institution by Cambodian people living in Japan and their view on health. J Jpn Soc Nurs Health Care. 2008;10: 55-64. https:// worldstm.com/Article/EF86461320008011000

13. Suh EE. The model of cultural competence through an evolutionary concept analysis. J Transcult Nurs. 2004;15: 93-102. https://doi.org/ 10.1177/1043659603262488.

14. McCarthy J, Cassidy I, Graham MM, Tuohy D. Conversations through barriers of language and interpretation. Br J Nurs. 2013;22(6): 335-339. https://doi.org/10.12968/bjon.2013.22.6.335.

15. Jirwe M, Gerrish K, Emami A. Student nurses' experiences of communication in cross-cultural care encounters. Scand J Caring Sci. 2010;24: 436–444. https://doi.org/

10.1111/j.1471-6712.2009. 00733.x.

16. Osmancevic S, Schoberer D, Lohrmann C, Großschädl F. Psychometric properties of used to measure the cultural competence of nurses: A systematic review. Int J Nurs Stud. 2021;113: 103789. https://doiorg/10.1016/j.ijnurstu.2020.103789

17. Shen Z. Cultural competence models and cultural competence assessment instruments in nursing: A literature review. J Transcult Nurs. 2015;26(3): 308-321. https://doi: 10.1177/1043659614524790.

18. Sugiura K. Survey-based analysis of cultural competence in nursing and its predictors: Comparison between nurses who were Japan overseas cooperation volunteers and nurses working at municipal hospitals. Jpn J Nurs Sci. 2003;3: 22-36. https://doi.org/10.5630/jans1981.23.3_22

19. Campinha-Bacote J. The process of cultural competence in the delivery of healthcare services: A model of care. J Transcult Nurs. 2002;13(3): 181-184. https:// doi.org.10.1177/10459602013003003

20. Mochizuki Y, Noji A. Essential points of cultural safety care from change of cultural care models. Health Sci. 2017;33(4): 245-254. https://jglobal.jst.go.jp/ detail? GLOBAL ID=201702287620586870

21. Nakanishi Y, Hirooka S, Yokoya S. Relationship between general motivation and social critical thinking: Competence for feeling interest and for thinking critically

among undergraduate students. Bulletin of the Integrated Center for Educational Research and Practice, Mie University. 2006; 26: 57-66. https://cir.nii.ac.jp/ crid/10102 82256857501957?lang=en.

22. Ogata Y. Study on factor contributing to the critical thinking disposition of nurses in social context. Bulletin of Hokkaido Bunkyo University. 2018;42: 63-72. https://do-bunkyodai.repo.nii.ac.jp/record/750/files/063074.尾形先生.pdf

23. Suzuki Y. Development of Intercultural Sensitivity scale in Japan. Bulletin of Graduate School of Human Development and Environment Kobe University. 2016;9(2): 39-44. https://cir.nii.ac.jp/crid/1390572174883505792.

24. Chen G, Starosta W. J. The development and validation of the Intercultural Sensitivity scale. H Comm. 2000;3: 1-15. https://psycnet.apa.org/record/2018-23164-001.

25. Arata Y, Nakao H, Hamada Y. The structure of motivation for growth of clinical nurses. J Jpn Acad Nurs Sci. 2019;39: 29–37. https://doi.org/10.5630/jans.39. 29

26. Bernhard G, Knibbe RA, von Wolff A, Dingoyan D, Schulz H, Mösko M. Development and psychometric evaluation of an instrument to assess cross-cultural competence of healthcare professionals (CCCHP). PLOS ONE. 2015;10(12): e0144049. https://doi.org/10.1371/journal.pone.0144049.

27. Halabi O. J, de Beer J. Exploring the cultural competence of undergraduate nursing students in Saudi Arabia. Nurse Educ Today. 2018;62: 9-15. https://doi.org/10.1016/j.nedt.2017.12.005

28. Olt. H, Jirwe M, Emmami A. psychometric evaluation of the Swedish adaptation of the Inventory for Assessing the Process of Cultural Competence Among Healthcare Professionals – Revised (IAPCC-R). J Transcult Nurs. 2009;21: 1. https://doi.org/10.1177/1043659609349064.

29. Markus HR, Kitayama S. Culture and the self: Implications for cognition, emotion, and motivation. Psychol Rev. 1991;98: 224-253. https://doi.org/10.1037/0033295X.98.2.224.

30. Hashimoto H. Interdependence as a self-sustaining set of beliefs. Jpn J Exp Soc Psychol. 2011;50 (2): 182-193. https://doi.org/10.2130/jjesp.50.182.

31. Nisbett RE. The geography of thought: How Asians and Westerners think differently… and why. Free Press; 2003.

32. Ministry of Land, Infrastructure, Transport and Tourism. Changes in Japanese sensibilities. Land Transport Whie Paper 2019. https://www.mlit.go.jp/hakusyo/mlit/h30/hakusho/r01/pdf/np101300.pdf.

33. Lin MH, Chang TH, Lee YuH, Wang PY, Lin LH, Hsu HC. Developing and validating the Nursing Cultural Competence Scale in Taiwan. PLOS ONE. 2019;14(8): e0220944. https://doi.org/10.1371/journal.pone.0220944

34. Ministry of Health, Labour and Welfare. Overview of health administration report cases (employed medical workers) for 2020. https://www.mhlw.go.jp/ toukei/saikin/ hw/ eisei/20/kekka1.pdf.

Click here to access/download
Supporting Information
Table 1.docx

Click here to access/download
Supporting Information
Table 2.docx

Click here to access/download
Supporting Information
Table 3 .docx

Click here to access/download
Supporting Information
Table 4 .docx

Click here to access/download
Supporting Information
Table 5 .docx

Click here to access/download
Supporting Information
Table 6 .docx

Developing and validating a cross-cultural competence scale for Japanese nurses

Masako Sakamoto[1], Shigemi Iriyama [1], Yu Mon Saw [2]

[1]Department of Integrated Health Sciences, Nagoya University Graduate school of Medicine, Nagoya, Japan,

[2] Department of Community and Global Health, University of Tokyo, Tokyo, Japan

ORCID ID: 0009-0002-0712-8798

E-mail: sakamoto.masako.w3@s.mail.nagoya- u.ac.jp (MS)

Short title: A cross-cultural competence scale for Japanese nurses

Abstract

In recent years, Japan has experienced a significant increase in the number of foreign students and workers entering the country. This has resulted in a vast number of international patients in medical facilities. This shift emphasizes the immediate need for Japanese nurses who are both clinically proficient and culturally attuned. In response, our research developed and validated the Cross-cultural Competence Scale for Japanese Nurses (CCCSJN) to better equip nurses for diverse patient care. We conducted a cross-sectional study in Japan's general hospitals using anonymous questionnaires with nurses and midwives. The scale, developed from data of 394 nurses, underwent both qualitative and quantitative evaluations to define its construct. We analyzed the data using exploratory factor analysis, criterion-related validity, internal consistency, and test-retest reliability, confirming the scale's reliability and validity. The exploratory analysis revealed five factors: "cross-cultural understanding," "cross-cultural communication skills," "motivation for cross-cultural nursing, "collaboration with multiple professions," and "respect for foreign patients." These factors explained 51.95% of the total variance. Cronbach's α for the CCCSJN was 0.92, and the test-retest reliability correlation was 0.77. The construct validity, criterion-related validity, internal consistency, and test-retest reliability of the CCCSJN were verified. The CCCSJN can be used to assess the cross-

cultural competencies of Japanese nurses and identify what skills need to be mastered, leading to improved cross-cultural competence and care.

Introduction

There has been a noticeable upward trend in the influx of foreign workers and students into Japan in recent years, resulting in an increase in the number of foreign patients seeking care in medical facilities. According to a Ministry of Health, Labour and Welfare report, the number of foreign patients per medical facility more than doubled between 2018 and 2020, from 24.3 to 50.2 [1]. In 2021, the number of medical facilities receiving foreign patients reached a record high, with 50.1% of all facilities responsible for accommodating this influx [1].

Despite the impact of the COVID-19 pandemic, the number of foreign residents has not decreased significantly, remaining at approximately 2.82 million in 2021 [2] or 2.5% of the overall population. Since April 29, 2023, restrictions on foreign entry into Japan have eased, international travel has increased, and the number of foreign patients visiting Japanese medical facilities is expected to increase. Medical facilities that previously had minimal or no contact with foreign patients must promptly establish a

system to accommodate them [3]. Similarly, there is an increasing demand for Japanese nurses who can effectively care for foreign patients.

Culturally sensitive care must be provided to effectively address the demands of foreign patients from various cultural backgrounds [4,5]. Although 72.5% of Japanese nurses have experience in caring for foreign patients [6], these nurses acknowledged that they have not been able to provide adequate care. Japanese nurses often experience anxiety and difficulty in language and communication [7–10], as well as difficulties in addressing cultural aspects such as lifestyle, diet, and religious practices [11,12].

To ensure the safe and uncomplicated delivery of medical care to foreign patients, nurses' competencies in interacting with culturally diverse individuals is critical. This goes beyond fundamental communicative skills, necessitating a sophisticated comprehension of various cultural factors such as lifestyle choices, dietary preferences, and religious practices [13]. Unfortunately, Japanese nurses are often hesitant to provide care to foreign patients because of the bewilderment caused by differences in communication, culture, and customs. A lack of cross-cultural competence can affect the quality of nursing care [14] and reduce patient satisfaction and safety [15]. However, if nurses can properly assess their own cross-cultural competence, they can

visualize what competencies are lacking. This increased self-awareness can improve the quality of care they provide to foreign patients.

Globally, numerous studies on nursing care for foreign patients have produced various scales to measure nurses' cross-cultural competence. Widely used scale concepts include three-dimensional models, such as the scale of beliefs and attitudes, knowledge, and skills developed by Suh et al. in the United States [13]. While several measures of cross-cultural competence have been properly tested and shown to be reliable, many lack the necessary validation [16, 17]. It is essential to employ appropriately validated scales to assess nurses' cross-cultural competencies.

Currently, only a few scales assess the cross-cultural competence of Japanese nurses. These include Cultural Competence in Nursing (CCN) [18] and the Japanese version of the Caffrey Cultural Competence Health Services (J-CCCHS) [6]. The CCN was first developed in Japan by Sugiura in 2003, referring to the Campinha–Bacote conceptual model [19,20]. Although construct validity was shown, the Cronbach's α for one factor was low (0.64). Additionally, the number of questions was large ($n = 46$), raising concerns about the burden on participants. Therefore, the validity and reliability of the scale needs further examination. Further, the J-CCCHS was developed through a large-scale survey conducted by Noji in 2017. Structural equation modeling was used for

the analysis, but the scale is not yet practical owing to insufficient model fit [6]. Further improvements are urgently needed to create a valid and reliable measure for Japanese nurses.

The study purpose was therefore to develop and validate the Cross-cultural Competence Scale for Japanese Nurses (CCCSJN).

Materials and methods

Preliminary study

To develop the CCCSJN, it was necessary to use questionnaire items that reflect the characteristics of Japanese nurses. Therefore, a preliminary qualitative study was conducted. The results were then used as items in the questionnaire.

We conducted 30-minute semi-structured interviews based on interviews with 14 Japanese nurses with experience in caring for foreign patients after obtaining informed consent. We analyzed the concept of cross-cultural responsiveness from data recorded by IC recorders and extracted eight categories: consideration for foreign patients, motivation to care for foreign patients, ingenuity in explanation, utilization of social resources, confirmation, dignity, financial support, and collaboration. We then drafted the 40-item CCCSJN.

To assess content validity, four university faculty members working as cross-cultural nursing researchers examined the clarity of each item and its relevance to the concept. Three items were removed because of duplication and ambiguity, leaving a final draft of 37 items in the aforementioned eight categories. The overall content validity index of the scale was 0.96.

Study design and study duration

We conducted a cross-sectional study using an anonymous self-administered questionnaire between August 2022 and January 2023.

Study participants

We examined nurses and midwives in general hospitals located in four cities in Aichi Prefecture with many foreign residents. The selection criteria for participants were nurses and midwives of Japanese nationality. Outpatient and ward nurses were excluded because administrative responsibilities greatly expanded their roles beyond simply providing care.

Sampling procedures and data collection

Japanese nurses were recruited through convenience sampling from six medical facilities with more than 200 beds. We selected Aichi Prefecture as the site for this study because it has the second-largest number of foreign residents after Tokyo. Consequently,

nurses working in Aichi Prefecture were speculated to have more experience caring for foreign patients than those in other prefectures. In total, there were 165 participating medical facilities. However, the number of eligible medical facilities was 27, excluding psychiatric, oncology, pediatric, and recuperation facilities and medical facilities with less than 200 beds. Research cooperation was requested in writing from the nursing directors of these 27 medical facilities, and consent was obtained from the nursing directors of six medical facilities. Nursing directors in the outpatient and ward departments distributed study request forms, two anonymous self-report questionnaires, and post-paid envelopes to the participants. We collected data by mail.

Definition of “cross-cultural competence”

“Cross-cultural competence” was operationally defined based on the three-dimensional model proposed by Suh et al. [13]. This term indicates the ability to provide appropriate nursing care for foreign patients from different cultural backgrounds. This ability requires cross-cultural knowledge, communication skills, and responding to foreign patients with respect.

Study measures

Social critical thinking orientation (SCTO)

To verify the criterion-related validity of the CCCSJN, we used two SCTO subscales—“understanding diversity” and “understanding others”—developed by Nakanishi et al. [21]. The SCTO comprises 27 items and seven factors: emphasis on logic and evidence, discrediting, understanding others, understanding diversity, authenticity, understanding key points, and determination. Permission to use the SCTO was obtained from the authors via email.

The subscales “understanding diversity” and “understanding others” correspond to the “power to understand people” and the “power to practice people-centered care” among the elements of nursing practical ability [22]. Therefore, the two subscales were adopted to infer that they were related to the cross-cultural ability of Japanese nurses to provide the necessary care to foreign patients. Both “understanding diversity” and “understanding others” comprise four items and are rated on a seven-point Likert scale from “not at all applicable” (1) to “very applicable” (7). We calculated the sum score, with higher scores indicating a higher degree of understanding diversity and others.

Japanese intercultural sensitivity scale (J-ISS)

To verify the criterion-related validity of the CCCSJN, we used a subscale of the J-ISS [23]: “positive feelings for different cultures.” The ISS was developed by Chen and Starosta [24], and the J-ISS was developed by Suzuki et al. [20]. The J-ISS comprises 22

items and three concepts: “positive feelings toward different cultures,” “ambivalent feelings toward different cultures,” and “negative feelings toward different cultures.” Permission to use the J-ISS was obtained from the authors via email.

“Positive feelings toward different cultures” comprises items with positive emotional content, such as actively wanting to know about cultural differences or enjoying cultural differences [23]. We speculated that this concept is related to cross-cultural competence needed by Japanese nurses caring for foreign patients and was adopted as the criterion-related validity of the CCCSJN. The J-ISS subscale “positive feelings toward different cultures” comprises 10 items with a five-point Likert scale ranging from “not at all” (1) to “very true” (5). We calculated the sum of the scores, with high scores indicating a high degree of positivity.

Socio-demographic characteristics

We collected socio-demographic information such as age, years of work experience, department affiliation, nursing qualifications, and educational background.

Cross-cultural experience in nursing

We collected data on the frequency of foreign patient care, experience of living abroad for more than a month, contact with foreigners, foreign language proficiency, and experience in cross-cultural education.

Statistical analysis

Age and years of nursing experience were not normally distributed and thus were calculated with median and interquartile range (IQR).

The construct validity of the CCCSJN was assessed using exploratory factor analysis. An item analysis was performed beforehand to examine ceiling and floor effects and item-total correlations. The principal factor method was selected for factor extraction and promax rotation was used because there was a correlation between the subscales. The statistical criteria determined the extraction of factors with eigenvalues of ≥ 1. Based on these analyses, items with loadings ≥ 0.40 were considered significant and factors were extracted.

To verify the criterion-related validity of the CCCSJN, the relationships between the subscales of the CCCSJN and the SCTO (“understanding others” and “understanding diversity”) and between the subscales of the CCCSJN and the J-ISS (“positive feelings toward different cultures”) were examined using Pearson’s correlation coefficients.

The reliability (internal consistency) of the CCCSJN was determined by Cronbach’s alpha coefficients. The test-retest reliability of the first and second surveys was indicated by intraclass correlation coefficients (ICCs).

Data were analyzed using SPSS version 22.0 (IBM SPSS Inc.).

Ethical considerations

We obtained ethical approval from the ethics committee of Nagoya University School of Medicine (no. 22-108) and the six medical facilities. The study purpose was fully explained to all participants, and participants were not obliged to participate in this study and could withdraw at any time. In order to consent to the research, a consent box was provided at the beginning of the questionnaire, and participants were asked to check the box if they consented.

Results

Descriptive statistics

In the first study, 406 out of 504 (72.2% effective response rate) responded by mail, and 394 out of 406 (97.0% effective response rate) responded to the CCCSJN questionnaire. All participants were women. The median age of the nurses was 33.0 years (IQR: 26–44), and their nursing experience was 9.8 years (IQR: 4.6–19.7). Of these, 330 (83.8%) completed their academic career at vocational schools and 51 (13.0%) had graduated from universities or graduate schools. There were 317 (80.5%) nurses working at city hospitals and 19 (4.8%) working at university hospitals (Table 1).

Table1 Japanese nurses' socio-demographic characteristics (N=394)

		n	%	Median	(IQR)
Age（years）				33	(26-44)
Length of clinical experience (years)				9.8	(4.6-19.7)
Qualification					
	Nurse	372	94.4		
	Midwife	22	5.6		
Educational background					
	Diploma	330	83.7		
	Bachelor's degree	44	11.2		
	Advanced diploma	9	2.3		
	Master's degree	7	1.8		
	Associate degree	4	1.0		
Department					
	Inpatient	321	81.5		
	Outpatient	73	18.5		
Number of nurses by employment facility					
	Municipal hospital	317	80.5		
	Consumers' co-operative hospital	46	11.7		
	University hospital	19	4.8		
	Public interest incorporated foundation hospital	12	3.0		

IQR=Interquartile range

https://doi.org/10.18999/2008709

There were 12 (3.0%) nurses who had at least one month's experience of living overseas, while five (1.3%) had overseas training or work experience. Fifty-four (13.7%) nurses had contact with foreigners other than patients in their daily lives, and 22 (5.6%) nurses had foreign skills equivalent to daily conversations. Ninety-five (24.2%) nurses had cross-cultural understanding and learning experiences, while 325 nurses (82.5%) had cared for one or more foreign patients in the past year. The median number of foreign patients in the past year were five (IQR: 3–10; Table 2).

Table 2 Japanese nurses' cross-cultural experience (N=394)

		n	%	Median	(IQR)
Stay abroad [a] (≧1month)	Yes	12	3.1		
	No	380	96.9		
Abroad nursing experience (Training/Work)	Yes	5	1.3		
	No	389	98.7		
Contact with foreigners other than foreign patients	Yes	54	13.7		
	No	340	86.3		
Conversational proficiency in a foreign language	Yes	22	5.6		
	No	372	94.4		
Cross-cultural learning [b]	Yes	95	24.2		
	No	298	75.8		
Experience of foreign patient care in the year	Yes	325	82.5		
	No	69	17.5		
Experience of foreign patient care in the year [c]				5	(3-10)

IQR= Interquartile range [a] Missing value：2 [b] Missing value :1 [c] Missing value：76

https://doi.org/10.18999/0002008753

Validity and reliability of the CCCSJN

CCCSJN construct validity

To confirm the construct validity of the CCCSJN, item analysis was performed before the exploratory factor analysis. The distribution showed a ceiling effect for Q19 (5.09), Q20 (5.01) and Q21 (5.08), while Q4 showed a floor effect (0.75). The I-T correlation was less than 0.30 for Q4 (0.06), Q9 (0.23), Q20 (0.29), and Q21 (0.21). Therefore, five of the 37 items were excluded from the CCCSJN(Table3).

Table 3 Item Analysis of the CCCSJN for Japanese nurses (N=394)

	Question items	Mean	SD	CE	FE	I-T
Q1	I treat foreign patients with respect.	4.02	0.72	4.74	3.30	0.56
Q2	I frequently talk to foreign patients.	3.40	0.90	4.30	2.50	0.60
Q3	I greet foreign patients in their native languages as much as possible.	2.68	1.11	3.79	1.57	0.38
Q4	When foreign patients are admitted to a ward, I always introduce them to the patients in the same room.	1.69	0.94	2.63	0.75	0.06
Q5	I try not to show foreign patients that I am in a hurry.	3.15	0.86	4.01	2.30	0.34
Q6	When I cannot satisfy the wishes of a foreign patient, I explain the reason and obtain his/her understanding.	3.54	0.84	4.38	2.70	0.53
Q7	When a foreign patient asks me a question about nursing care, I provide a detailed explanation.	3.61	0.80	4.42	2.81	0.58
Q8	I listen to foreign patients carefully and try to understand their thoughts.	3.86	0.70	4.57	3.16	0.66
Q9	When I know I'll be dealing with foreign patients in advance, I try to research the culture of the country they come from.	2.22	1.03	3.25	1.19	0.23
Q10	I try to provide the same care to foreign patients as I do to Japanese patients.	4.11	0.77	4.88	3.34	0.51
Q11	I apply the experiences I have learned from foreign patients in my nursing care.	3.16	0.86	4.02	2.31	0.52
Q12	When there is a difference between the wishes of foreign patients and the rules of the hospital, I think of other ways to address the issue together with the patient.	2.87	0.92	3.78	1.95	0.47
Q13	I want to be actively involved in the care of foreign patients.	2.87	0.96	3.83	1.91	0.49
Q14	I take time to explain matters to foreign patients.	3.45	0.85	4.30	2.60	0.63
Q15	I speak slowly to foreign patients in simple Japanese.	4.13	0.65	4.77	3.49	0.45
Q16	I use gestures to explain things to foreign patients who do not understand Japanese.	4.27	0.67	4.94	3.60	0.52
Q17	I explain to foreign patients by showing them illustrations and drawings.	3.44	1.01	4.45	2.43	0.35
Q18	I use a comparison chart for foreign patients in their native language or in an understandable foreign language and Japanese.	3.49	1.12	4.61	2.37	0.36
Q19	I use translation devices and translation apps for foreign patients.	4.10	0.99	5.09	3.11	0.34
Q20	I get the cooperation of family and friends who understand Japanese.	3.99	1.02	5.01	2.97	0.29
Q21	I always use medical interpreters when I give IC or important explanations to foreign patients.	3.99	1.09	5.08	2.90	0.21
Q22	I check the thoughts and intentions of foreign patients before providing care.	3.45	0.87	4.32	2.58	0.58
Q23	I observe the facial expressions and reactions of foreign patients to see if they understand what I want to convey.	4.06	0.63	4.68	3.44	0.66
Q24	I ask questions to make sure foreign patients understand what I have tried to convey.	3.72	0.81	4.53	2.91	0.39
Q25	I understand that the cultures of foreign patients may make them have different views regarding health and disease matters.	3.52	0.88	4.40	2.64	0.62
Q26	I understand that different cultures have different values.	3.89	0.77	4.66	3.12	0.69
Q27	I understand that foreign patients' religion or beliefs may dictate traditional diets or dietary restrictions.	4.03	0.73	4.76	3.30	0.69
Q28	I understand that foreign patients may have behavioral restrictions or rules according to their religion.	3.95	0.74	4.69	3.21	0.69
Q29	I understand that culturally sensitive nursing care must be provided to all foreign patients.	3.92	0.77	4.69	3.15	0.70
Q30	I understand that foreign patients who are here for a short period of stay may feel anxious about their hospital stay.	3.88	0.82	4.71	3.06	0.68
Q31	I understand that the health care system in foreign patients' country of origin is different.	3.50	0.94	4.44	2.56	0.61
Q32	I understand that foreign patients may have financial concerns.	3.79	0.78	4.57	3.01	0.63
Q33	I share information with the staff in my office in order to understand foreign patients. .	3.60	0.84	4.44	2.76	0.63
Q34	I have detailed information on the daily lives of foreign patients in my medical records.	3.15	0.85	4.00	2.30	0.52
Q35	I do not handle foreign patients by myself, I collaborate with my colleagues in the office.	3.79	0.75	4.54	3.04	0.62
Q36	I cooperate with staff from various professions to meet the wishes of foreign patients.	3.75	0.75	4.51	3.00	0.59
Q37	With the consent of the foreign patient, I share information with supporters.	3.55	0.81	4.36	2.74	0.54

CE=ceiling effect; FE=floor effect; I-T=i-t correlation

https://doi.org/10.18999/2008754

In the first exploratory factor analysis, Q5, Q18, Q22, and Q24 were excluded because their factor loadings were less than 0.4. The factor loading for Q8 was 0.38, but it was borderline and similar to the factor concepts in questions 6 and 7; therefore, it was not excluded and was reserved. In the second exploratory factor analysis, all questionnaire items were above 0.40. The factor loading of Q17 was 0.65, but it was excluded because it was one factor and one item (Table 4). To ensure that exclusion criterion of less than 0.40 was reasonable, an exploratory factor analysis was performed by excluding items with a factor loading of less than 0.35. The result was a six-factor structure with 29 items. However, one factor included only two items: Q17 and Q18. Cronbach's α was low as 0.53.

Table 4 Factor loadings on CCCSJN for Japanese nurses after items analysis (N=394)

32 Items	1	2	3	4	5	6
Q26. I understand that different cultures have different values	**0.86**	0.11	0.03	-0.11	-0.12	0.05
Q28. I understand that foreign patients may have behavioral restrictions or rules according to their religion.	**0.86**	-0.07	0.19	-0.11	-0.03	0.00
Q27. I understand that foreign patients' religion or beliefs may dictate traditional diets or dietary restrictions	**0.82**	-0.07	0.22	-0.11	-0.04	-0.01
Q31. I have detailed information on the daily lives of foreign patients in my medical records.	**0.76**	0.06	-0.27	0.14	0.00	-0.10
Q29. I understand that culturally sensitive nursing care must be provided to all foreign patients.	**0.75**	-0.08	0.12	0.03	0.06	-0.04
Q32. I understand that foreign patients may have financial concerns.	**0.69**	-0.07	-0.13	0.19	0.11	-0.14
Q25. I understand that the cultures of foreign patients may make them have different views regarding health and disease matters.	**0.65**	0.28	-0.20	0.00	-0.11	0.13
Q30. I understand that foreign patients who are here for a short period of stay may feel anxious about their hospital stay.	**0.64**	-0.13	0.04	0.09	0.13	0.09
Q13. I want to be actively involved in the care of foreign patients.	-0.04	**0.85**	0.04	-0.04	-0.21	0.03
Q2. I frequently talk to foreign patients.	-0.15	**0.62**	0.25	0.04	0.03	-0.15
Q3. I greet foreign ptients in their native languages as much as possible.	-0.03	**0.53**	-0.07	-0.10	0.03	0.14
Q11. I apply the experiences I have learned from foreign patients in my nursing care.	0.15	**0.49**	-0.09	-0.01	-0.03	0.10
Q12. When there is a difference between the wishes of foreign patients and the rules of the hospital, I think of other ways to come to terms with them together with foreign patients.	0.07	**0.44**	-0.20	0.06	0.15	0.14
Q14. I take time to explain to foreign patients.	0.11	**0.43**	0.17	0.00	0.10	0.01
Q22. I check the thoughts and intentions of foreign patients before providing care.	0.07	0.07	0.07	0.07	0.07	0.07
Q24. I ask questions to make sure foreign patients understand what I have tried to convey.	0.26	0.28	0.13	0.07	0.11	0.00
Q15. I speak slowly to foreign patients in simple Japanese.	-0.06	-0.10	**0.75**	0.00	0.01	0.07
Q16. I use gestures to explain things to foreign patients who do not understand Japanese.	0.12	-0.11	**0.69**	0.04	-0.09	0.19
Q1. I treat foreign patients with respect.	-0.07	0.30	**0.58**	0.11	-0.14	-0.15
Q10. I try to provide the same care to foreign patients as I do to Japanese patients.	-0.01	0.08	**0.53**	-0.02	0.12	-0.09
Q23. I observe the facial expressions and reactions of foreign patients to see if they understand what I want to convey.	0.33	-0.03	**0.41**	0.03	0.06	0.04
Q37. With the consent of the foreign patient, I share information with supporters.	0.03	-0.06	0.01	**0.78**	-0.12	0.15
Q36. I cooperate with staff from various professions to meet the wishes of foreign patients.	-0.09	-0.09	0.12	**0.71**	0.06	0.13
Q33. I share information with staff in my office in order to understand foreign patients.	0.25	0.04	-0.06	**0.68**	-0.09	-0.15
Q35. I do not handle foreign patients by myself, I collaborate with my colleagues in the office.	0.02	-0.09	0.21	**0.65**	0.00	-0.06
Q34. I have detailed information on the daily lives of foreign patients in my medical records.	-0.08	0.20	-0.15	**0.53**	0.10	0.10
Q7. When a foreign patient asks me a question about nursing care, I provide a detailed explanation.	0.03	-0.01	-0.03	-0.09	**0.92**	-0.08
Q6. When I cannot satisfy the wishes of a foreign patient, I explain the reason and obtain his/her understanding.	-0.07	-0.11	-0.01	0.05	**0.80**	0.08
Q8. I listen to foreign patients carefully and try to understand their thoughts.	0.01	0.22	0.28	-0.03	**0.38**	0.08
Q5. I try not to show foreign patients that I am in hurry.	0.02	0.22	0.00	-0.05	0.29	-0.05
Q17. I explain to foreign patients by showing them illustrations and drawings.	-0.05	0.13	0.02	0.08	-0.01	**0.65**
Q18. I use a comparison chart for foreign patients in their native language or in an understandable foreign language and Japanese.	0.00	0.12	0.12	0.11	-0.01	0.36
Eigen values	10.33	1.91	1.36	1.08	0.81	0.65
Percentage of variance	32.29	5.98	4.25	3.37	2.53	2.05
Cumulative of total variance explained	32.29	38.27	42.52	45.89	48.42	50.46

https://doi.org/10.18999/2008755

A third exploratory factor analysis was performed, and the criterion was less than 0.40. Finally, a five-factor structure comprising 27 items was adopted. The model fit showed that the validity of the Kaiser–Meyer–Olkin sample was 0.918, and Bartlett's sphericity test showed a good fit ($p < 0.001$). The cumulative contribution rate was 51.95 (Table 5).

Table 5 Factor loadings on CCCSJN for Japanese Nurses (N=394)

27 items α=0.92	1	2	3	4	5
**Foctor1：Understanding of cross- cultural （α=0.91）**					
Q28. I understand that foreign patients may have behavioral restrictions or rules according to their religion.	**0.85**	0.20	-0.07	-0.11	-0.04
Q26. I understand that different cultures have different values	**0.85**	0.04	0.10	-0.08	-0.11
Q27. I understand that foreign patients'religion or beliefs may dictate traditional diets or dietary restrictions	**0.82**	0.23	-0.06	-0.12	-0.05
Q31. I have detailed information on the daily lives of foreign patients in my medical records.	**0.74**	0.13	-0.07	0.00	0.05
Q29. I understand that culturally sensitive nursing care must be provided to all foreign patients.	**0.73**	-0.28	0.08	0.11	0.01
Q32. I understand that foreign patients may have financial concerns.	**0.67**	-0.11	-0.03	0.12	0.11
Q25. I understand that cultures of foreign patients may have different views regarding health and disease matters.	**0.65**	-0.19	0.26	0.04	-0.08
Q30. I understand that foreign patients with a short period of stay may feel anxous about their hospital stay.	**0.63**	0.04	-0.11	0.11	0.14
**Factor2：Cross-cultural communication ability （α=0.78）**					
Q15. I speak slowly to foreign patients in simple Japanese.	-0.06	**0.76**	-0.10	0.03	0.00
Q16. I use gestures to explain things to foreign patients who do not understand Japanese.	0.12	**0.67**	-0.11	0.10	-0.05
Q1. I treat foreign patients with respect.	-0.07	**0.58**	0.30	0.04	-0.12
Q10. I try to provide the same care to foreign patients as I do to Japanese patients.	-0.01	**0.53**	0.09	-0.05	0.12
Q23. I observe the facial expressions and reactions of foreign patients to see if they understand what I want to convey.	0.33	**0.41**	-0.05	0.06	0.04
**Factor3：Motivation for cross-cultural nursing （α=0.75）**					
Q13. I want to be actively involved in the care of foreign patients.	-0.02	0.07	**0.82**	-0.03	-0.17
Q2. I frequently talk to foreign patients.	-0.13	0.27	**0.59**	-0.01	0.04
Q3. I greet foreign patients in their native languages as much as possible.	-0.01	-0.06	**0.47**	-0.02	0.04
Q11. I apply the experiences I have learned from foreign patients in my nursing care.	0.17	-0.08	**0.47**	0.03	0.00
Q12. When there is a difference between the wishes of foreign patients and the rules of the hospital, I think of other ways to address the issue together with the patient.	0.10	-0.18	**0.42**	0.10	0.15
Q14. I take time to explain matters to foreign patients.	0.12	0.19	**0.41**	0.01	0.12
**Factor4：Cooporation with multiple professions （α=0.82）**					
Q37. With the consent of the foreign patient, I share information with supporters.	0.02	0.02	-0.04	**0.81**	-0.10
Q36. I cooperate with staff from various professions to meet the wishes of foreign patients.	-0.08	0.14	-0.06	**0.71**	0.06
Q35. I do not handle foreign patients by myself alone, but collaborate with my colleagues in the office.	0.01	0.23	-0.04	**0.60**	-0.01
Q33. I share information closely with staff in my office in order to understand foreign patients. .	0.23	-0.04	0.07	**0.58**	-0.08
Q34. I have detailed information on the daily lives of foreign patients in my medical records.	-0.06	-0.13	0.20	**0.54**	0.11
**Factor5：Respect for foreign patiens （α=0.79）**					
Q7. When a foreign patient asks me a question about nursing care, I explain the reason in detail.	0.03	-0.03	0.02	-0.11	**0.93**
Q6. When I cannot satisfy the wishes of a foreign patient, I explain the reason and obtain his/her understanding.	-0.05	0.03	-0.07	0.11	**0.69**
Q8. I listen to foreign patients carefully and try to understand their thoughts.	0.03	0.27	0.22	-0.01	**0.42**
Eigen values	9.17	1.76	1.34	0.99	0.76
Percentage of variance	33.98	6.53	4.95	3.66	2.83
Cumulative of total variance axplained	33.98	40.51	45.46	49.12	51.95

https://doi.org/10.18999/2008756

The first factor contributed 33.98%, the second factor 6.53%, the third factor 4.95%, the fourth factor 3.66%, and the fifth factor 2.83%. The factor loadings were

0.63–0.85 for eight items on the first factor, 0.41–0.76 for five items on the second factor, 0.41–0.82 for six items on the third factor, 0.55–0.81 for five items on the fourth factor, and 0.42–0.93 for three items on the fifth factor. We named the first factor “cross-cultural understanding,” the second factor “cross-cultural communication skills,” the third factor “motivation for cross-cultural nursing,” the fourth factor “collaboration with multiple professions,” and the fifth factor “respect for foreign patients.”

Criterion-related validity of the CCCSJN

The total CCCSJN score showed a significant positive correlation with the SCTO subscales “understanding diversity” and “understanding others” (understanding diversity: $r = 0.51$, $p < .001$; understanding others: $r = 0.47$, $p < .001$). The total score of CCCSJN also showed a significant positive correlation with the J-CCSS subscale “positive feelings toward different cultures” ($r = 0.61$, $p < .001$; Table 6).

Table 6 Correlation between total scores of the CCCSJN and two subscales of the SCTO, a subscale of J-ISS

N=390

	CCCSJN	
	r	p
1. The Social Critical Thinking Orientation: subscale **diversity understanding**	0.51	＜.001
2. The Social Critical Thinking Orientation: subscale **other understanding**	0.47	＜.001
3. Japanese version of the Intercultural Sensitivity Scale: subscale **positive feelings toward different cultures**	0.61	＜.001

r = Pearson correlation coefficient p = probability value

https://doi.org/10.18999/2008757

Internal consistency of CCCSJN

The Cronbach's α for the overall CCCSJN was 0.92 (Table 5). Cronbach's α for each subscale of CCCSJN were 0.91 for "cross-cultural understanding," 0.78 for "cross-cultural communication skills," 0.75 for "motivation for cross-cultural nursing," 0.82 for "collaboration with multiple professions," and 0.79 for "respect for foreign patients." There was a strong correlation between the CCCSJN total score and all subscale factors (rs = 0.70–0.84, p < .001). Moderate correlations existed between each subscale (rs = 0.44–0.56, p < .001).

CCCSJN test-retest reliability

Of the 394 participants, 341 returned the questionnaire in the second study (response rate = 60.8%). Substantial correlations were found between the first and second studies (ICC [1,1] = 0.767 [0.72–0.81], p < .001).

Discussion

This study developed a scale—the CCCSJN—to measure the cross-cultural capacity of Japanese nurses to care for foreign patients. The scale demonstrated construct validity, criterion-related validity, internal consistency, and test-retest reliability. The CCCSJN had a five-factor structure, in which the third, fourth, and fifth factors reflected

the cultural characteristics of Japanese nurses. The third factor, “motivation for cross-cultural nursing,” was the concept of nurses learning about cultural diversity and being positive about actively engaging with patients from different cultural backgrounds. In a previous study [25], a significant positive association was found between work attitude and motivation. Thus, it can be inferred that the concept of “motivation for cross-cultural nursing” promotes the ability of Japanese nurses to cope with cross-cultural situations. In Germany, a scale expressed as the concept of motivation and curiosity was developed [26], validated, and was deemed sufficiently reliable for adoption as a high-impact primary factor. This concept is also similar to the “cultural desires” of Campinha–Bacote’s conceptual model. However, only a few scales adopted the concept of “cultural desires” [27,28], and the validity and reliability were poorly tested.

The fourth factor, “collaboration with multiple professions,” was not represented by existing scales in Europe, the United States, and Japan, confirming that this concept is unique to the CCCSJN. This means that nurses do not have to manage foreign patients on their own but can share information closely with staff at their workplace and collaborate with a wide range of professionals to ensure that they respond to the needs of foreign patients. Compared to the West, Japanese culture favors a mutually cooperative self-view [29,30], valuing cooperative relationships

between individuals [31]. Japanese people are also more interested in group goals and cooperative behavior. Although “collaboration with multiple professions” is essential for nurses, it was particularly evident in the CCCSJN.

The fifth factor, “respect for foreign patients,” refers to the attitude of responding to foreign patients with respect for their wishes and intentions. The Japanese are often characterized as having a national character that emphasizes respect for others, values compassion and courtesy, and may prioritize the needs of others over their own. In a 2019 Ministry of Land, Infrastructure, Transport, and Tourism attitude survey, more than 80% of respondents said it was important to be respectful and considerate of others [32]. “Respect for foreign patients” was an important attitude for nurses and a concept that emerged prominently in the CCCSJN.

“Use of social resources” was not included in the five-factor CCCSJN, although it was suggested that many Japanese nurses use social resources. “Use of a translation device,” “cooperation with family and friends of patients who could understand Japanese,” and “use of medical interpreters” were excluded at an early stage because of the ceiling effect. This meant that many Japanese nurses scored the highest.

Proof of criterion-related validity suggests that the CCCSJN could be an

appropriate measure of cross-cultural competence. Total CCCSJN score was significantly positively correlated with the SCTO subscales “understanding diversity” and “understanding others.” These questions were similar to those in the first factor of the CCCSJN, “cross-cultural understanding,” and were useful for understanding foreign patients. Japanese nurses also tended to emphasize “understanding diversity” and “understanding others” even in their nursing practice for Japanese patients [22]. The total CCCSJN score also showed a significant positive correlation with the J-CCSS subscale “positive feelings toward different cultures,” which was related to flexibility and adaptability in cross-cultural situations, and meaningful communication inspired by responses from others [23].

The CCCSJN had high internal consistency. Specifically, the Cronbach’s α for all CCCSJN items was high at 0.92, and the five subscales also showed high internal consistency at 0.75–0.91, indicating high reliability. The other scales showed high internal consistency, similar to that of the CCCSJN. The J-CCCHS showed Cronbach’s α for the five subscales ranged from 0.76–0.90 [6]. The Nursing Cultural Competence Scale had Cronbach’s α for the four-subscale that ranged from 0.82–0.84 [33]. Moderate to strong correlations between the CCCSJN and subscales was also obtained (rs = 0.68–0.84, p < .001). Additionally, a strong positive correlation was found

between the first and second studies on the test-retest reliability of the CCCSJN, indicating the stability of the scale.

Study limitations

This study has three limitations. The first limitation is selection bias because we recruited at medical facilities in which foreign patients are common. A multistage sampling study of 5430 nurses nationwide in Japan found that 72.5% had experienced caring for foreign patients [6], compared with 82.5% in this study. It is difficult to generalize the current findings to all Japanese nurses because this may not apply to those working in facilities in areas where there are few foreign residents. However, the current results could be applicable to nurses in urban areas, such as the Kanto and Kansai regions.

Second, recall bias could have occurred because some questions required recalling the content of care for foreign patients and the number of times they administered such care in a year. The recall period must be considered to avoid memory ambiguity and ensure accurate answers.

Third, female nurses were the only participants. The percentage of male nurses in Japan is 8.1%, which is about two times higher than 10 years ago [34], and this trend is expected to continue. Future studies including male nurses are warranted.

Conclusions

This study was the first to use a semi-structured interview method to develop a scale of cross-cultural competence for Japanese nurses caring for foreign patients. The scale has a five-factor structure and demonstrated construct validity, criterion-related validity, internal consistency, and test-retest reliability. Therefore, the scale is useful for assessing cross-cultural competence. The CCCSJN can be employed to assess the cross-cultural competencies of Japanese nurses and identify those that need to be mastered, leading to improved cross-cultural competencies and care.

Acknowledgments

We express our sincere appreciation to all the Japanese nurse from the six medical facilities in Aichi, Japan for their kind support and active cooperation in this study.

Authors' contributions

Conceptualization: Masako Sakamoto, Shigemi Iriyama, Yu Mon Saw

Data Curation: Masako Sakamoto, Shigemi Iriyama

Formal Analysis: Masako Sakamoto, Shigemi Iriyama, Yu Mon Saw

Funding acquisition: Masako Sakamoto

Investigation: Masako Sakamoto, Shigemi Iriyama, Yu Mon Saw

Methodology: Masako Sakamoto, Shigemi Iriyama, Yu Mon Saw

Project Administration: Masako Sakamoto, Shigemi Iriyama

Resources: Masako Sakamoto

Supervision: Masako Sakamoto, Shigemi Iriyama, Yu Mon Saw

Validation: Masako Sakamoto, Shigemi Iriyama

Visualization: Masako Sakamoto, Shigemi Iriyama

Writing – original draft: Masako Sakamoto, Shigemi Iriyama, Yu Mon Saw

Writing – review & editing: Masako Sakamoto, Shigemi Iriyama, Yu Mon Saw

References

1. Ministry of Health, Labour and Welfare. Reiwa actual situation regarding the acceptance of foreign patients at medical institutions in the third year. https://www.mhlw.go.jp/stf/newpage_25805.html.

2. Immigration Services Agency. Number of foreign residents as of June 2021. https://www.moj.go.jp/isa/publications/press/1300017.html

3. Ministry of Health, Labour and Welfare. Fact-finding Survey on the Acceptance of f foreign patients at Medical Institutions Result Report. https://www.mhlw.go.jp/content/ 10800000/000940326.pdf.

4. International Association of Nurses (ICN). Code of Ethics for ICN Nurses (2021 Edition). https://www.nurseor.jp/nursing/international/icn/document/ethics /index.html.

5. Japan Nursing Association: Code of Ethics for Nurses. https://www.nurse.or.jp/ nursing/practice/rinri/rinri.html.

6. Noji A, Mochizuki Y, Nosaki A, Glaser D, Gonzales L, Mizobe A, et al. Evaluating cultural competence among Japanese clinical nurses: Analyses of a translated scale. Int J Nurs Pract. 2017;23: Suppl 1. https://doi.org/10.1111/ijn.12551

7. Inoue C, Matsui M, Lee S, Nakamura N, Minoura S, Ushijima H. Research into the problems faced by and support given to non-Japanese speakers in need of perinatal care: From an analysis of the deliveries in one hospital over a 12-year period. J Int Health. 2006;21(1): 25-32. https://www.researchgate.net/publication/344014266

8. Iwata M, Yuki H. A literature review of the challenges and measures to promote cross-cultural communication in nursing practice. Journal of Japanese Society for International Nursing. 2021;(2): 12-22. https://www.jsin.jp/PDF/2021-2_paperNo2ENG. Pdf

9. Maeno M, Sakuyama M, Motoyama S, Matsuo H. Japanese nurses' view of perioperative management of foreign patients in Osaka. J Int Health. 2011;26(4): 272-280. https://doi.org/10.11197/JAIH.26.273

10. Nonaka C, Higuchi M. The process of building relationships between foreign patients and nurses in Japan. J Int Health. 2010;25(1): 21-32. https://doi.org/10.11197/jaih.25.21.

11. Teraoka M, Muranaka Y. Aspects of cross-cultural experience perceived by foreigners living in Japan when using its healthcare services. J Jpn Acad Nurs Sci. 2017;37: 35-44. https://doi.org/10.5630/jans.37.35.

12. Itoi Y. Feeling with the use of medical institution by Cambodian people living in Japan and their view on health. J Jpn Soc Nurs Health Care. 2008;10: 55-64. https:// worldstm.com/Article/EF86461320008011000

13. Suh EE. The model of cultural competence through an evolutionary concept analysis. J Transcult Nurs. 2004;15: 93-102. https://doi.org/ 10.1177/1043659603262488.

14. McCarthy J, Cassidy I, Graham MM, Tuohy D. Conversations through barriers of language and interpretation. Br J Nurs. 2013;22(6): 335-339. https://doi.org/10.12968/bjon.2013.22.6.335.

15. Jirwe M, Gerrish K, Emami A. Student nurses' experiences of communication in cross-cultural care encounters. Scand J Caring Sci. 2010;24: 436–444. https://doi.org/

10.1111/j.1471-6712.2009. 00733.x.

16. Osmancevic S, Schoberer D, Lohrmann C, Großschädl F. Psychometric properties of used to measure the cultural competence of nurses: A systematic review. Int J Nurs Stud. 2021;113: 103789. https://doiorg/10.1016/j.ijnurstu.2020.103789

17. Shen Z. Cultural competence models and cultural competence assessment instruments in nursing: A literature review. J Transcult Nurs. 2015;26(3): 308-321. https://doi: 10.1177/1043659614524790.

18. Sugiura K. Survey-based analysis of cultural competence in nursing and its predictors: Comparison between nurses who were Japan overseas cooperation volunteers and nurses working at municipal hospitals. Jpn J Nurs Sci. 2003;3: 22-36. https://doi.org/10.5630/jans1981.23.3_22

19. Campinha-Bacote J. The process of cultural competence in the delivery of healthcare services: A model of care. J Transcult Nurs. 2002;13(3): 181-184. https:// doi.org.10.1177/10459602013003003

20. Mochizuki Y, Noji A. Essential points of cultural safety care from change of cultural care models. Health Sci. 2017;33(4): 245-254. https://jglobal.jst.go.jp/ detail? GLOBAL ID=201702287620586870

21. Nakanishi Y, Hirooka S, Yokoya S. Relationship between general motivation and social critical thinking: Competence for feeling interest and for thinking critically

among undergraduate students. Bulletin of the Integrated Center for Educational Research and Practice, Mie University. 2006; 26: 57-66. https://cir.nii.ac.jp/ crid/10102 82256857501957?lang=en.

22. Ogata Y. Study on factor contributing to the critical thinking disposition of nurses in social context. Bulletin of Hokkaido Bunkyo University. 2018;42: 63-72. https://do-bunkyodai.repo.nii.ac.jp/record/750/files/063074.尾形先生.pdf

23. Suzuki Y. Development of Intercultural Sensitivity scale in Japan. Bulletin of Graduate School of Human Development and Environment Kobe University. 2016;9(2): 39-44. https://cir.nii.ac.jp/crid/1390572174883505792.

24. Chen G, Starosta W. J. The development and validation of the Intercultural Sensitivity scale. H Comm. 2000;3: 1-15. https://psycnet.apa.org/record/2018-23164-001.

25. Arata Y, Nakao H, Hamada Y. The structure of motivation for growth of clinical nurses. J Jpn Acad Nurs Sci. 2019;39: 29–37. https://doi.org/10.5630/jans.39. 29

26. Bernhard G, Knibbe RA, von Wolff A, Dingoyan D, Schulz H, Mösko M. Development and psychometric evaluation of an instrument to assess cross-cultural competence of healthcare professionals (CCCHP). PLOS ONE. 2015;10(12): e0144049. https://doi.org/10.1371/journal.pone.0144049.

27. Halabi O. J, de Beer J. Exploring the cultural competence of undergraduate nursing students in Saudi Arabia. Nurse Educ Today. 2018;62: 9-15. https://doi.org/10.1016/j.nedt.2017.12.005

28. Olt. H, Jirwe M, Emmami A. psychometric evaluation of the Swedish adaptation of the Inventory for Assessing the Process of Cultural Competence Among Healthcare Professionals – Revised (IAPCC-R). J Transcult Nurs. 2009;21: 1. https://doi.org/10.1177/1043659609349064.

29. Markus HR, Kitayama S. Culture and the self: Implications for cognition, emotion, and motivation. Psychol Rev. 1991;98: 224-253. https://doi.org/10.1037/0033295X.98.2.224.

30. Hashimoto H. Interdependence as a self-sustaining set of beliefs. Jpn J Exp Soc Psychol. 2011;50 (2): 182-193. https://doi.org/10.2130/jjesp.50.182.

31. Nisbett RE. The geography of thought: How Asians and Westerners think differently… and why. Free Press; 2003.

32. Ministry of Land, Infrastructure, Transport and Tourism. Changes in Japanese sensibilities. Land Transport Whie Paper 2019. https://www.mlit.go.jp/hakusyo/mlit/h30/hakusho/r01/pdf/np101300.pdf.

33. Lin MH, Chang TH, Lee YuH, Wang PY, Lin LH, Hsu HC. Developing and validating the Nursing Cultural Competence Scale in Taiwan. PLOS ONE. 2019;14(8): e0220944. https://doi.org/10.1371/journal.pone.0220944

34. Ministry of Health, Labour and Welfare. Overview of health administration report cases (employed medical workers) for 2020. https://www.mhlw.go.jp/ toukei/saikin/ hw/ eisei/20/kekka1.pdf.

Response to Reviewers

Manuscript ID; PONE-D-23-34062

We sincerely appreciate your comments on our manuscript.
We have revised and improved our manuscript as suggested.
In the following, we provide a point-to-point response to every question raised. All answers are represented in red.

Reviewer#1

Please ensure that your manuscript meets PLOS ONE's style requirements, including those for file naming. The PLOS ONE style templates can be found at https://journals.plos.org/plosone/s/file?id=wjVg/PLOSOne_formatting_sample_main_body.pdf and https://journals.plos.org/plosone/s/file?id=ba62/PLOSOne_formatting_sample_title_authors_affiliations.pdf.

Response: We took the Plos one style template as a reference, double-spaced to my manuscript and numbered each line. And we have inserted six tables after the first paragraph of the relevant paper content.

Reviewer#2

In your Data Availability statement, you have not specified where the minimal data set underlying the results described in your manuscript can be found. PLOS defines a study's minimal data set as the underlying data used to reach the conclusions drawn in the manuscript and any additional data required to replicate the reported study findings in their entirety. All PLOS journals require that the minimal data set be made fully available. For more information about our data policy, please see http://journals.plos.org/plosone/s/data-availability.

""Upon re-submitting your revised manuscript, please upload your study's minimal underlying data set as either Supporting Information files or to a stable, public repository and include the relevant URLs, DOIs, or accession numbers within your revised cover letter. For a list of acceptable repositories, please see http://journals.plos.org/plosone/s/data-availability#loc-recommended-repositories. Any potentially identifying patient information must be fully anonymized.

Important: If there are ethical or legal restrictions to sharing your data publicly, please explain these restrictions in detail. Please see our guidelines for more information on what we consider unacceptable restrictions to publicly sharing data: http://journals.plos.org/plosone/s/data-availability#loc-unacceptable-data-access-restrictions. Note that it is not acceptable for the authors to be the sole named individuals responsible for ensuring data access.

We will update your Data Availability statement to reflect the information you provide in your cover letter.

Response: The dataset, which is the minimal basis for the study, was uploaded as a public repositor by the Nagoya University Repository.

The uploaded data set is as follows.

Table 1 https://doi.org/10.18999/2008709

Table 2 https://doi.org/10.18999/0002008753

Table 3 https://doi.org/10.18999/2008754

Table 4 https://doi.org/10.18999/2008755

Table 5 https://doi.org/10.18999/2008756

Table 6 https://doi.org/10.18999/2008757

Reviewer#3

PLOS requires an ORCID iD for the corresponding author in Editorial Manager on papers submitted after December 6th, 2016. Please ensure that you have an ORCID iD and that it is validated in Editorial Manager. To do this, go to 'Update my Information' (in the upper left-hand corner of the main menu), and click on the Fetch/Validate link next to the ORCID field. This will take you to the ORCID site and allow you to create a new iD or authenticate a pre-existing iD in Editorial Manager. Please see the following video for instructions on linking an ORCID iD to your Editorial Manager account:https://www.youtube.com/watch?v=_xcclfuvtxQ".

Response: I got ORCID ID. The number is 0009-0002-0712-8798.

Reviewer#4

Your ethics statement should only appear in the Methods section of your manuscript. If your ethics statement is written in any section besides the Methods, please move it to the Methods section and delete it from any other section. Please ensure that your ethics statement is included in your manuscript, as the ethics statement entered into the online submission form will not be published alongside your manuscript.

Response: We have confirmed that the statement of ethics is included in the methods of the

m a n u s c r i p t . The ethics statement entered in the online submission form was included in the manuscript. We slightly corrected the sentence in red.

Reviewer#4

Please include your tables as part of your main manuscript and remove the individual files. Please note that supplementary tables (should remain/ be uploaded) as separate ""supporting information"" files".

Response: We included the table as part of the manuscript and removed the individual files. We created the supplementary tables as separate "Supporting Information" files.

In addition, we reviewed the manuscript and revised the background and methodology to make the abstract more understandable. We Added missing information and fixed incorrect wording. The modifications are shown in red.

I checked the journal requirements and added or corrected them where necessary. Please let me know if there are any shortcomings or mistakes.
I am looking forward to hearing from you.

Kind regards,
Masako Sakamoto (PONE-D-23-34062)
