# Supplementary material for: Developing and validating a cross-cultural competence scale for Japanese nurses
Source: PLoS One. 2025 Jan 9;20(1):e0310650. doi: 10.1371/journal.pone.0310650 (PMC11717215; doi:10.1371/journal.pone.0310650)
Supplement: Attachment — Submitted filename: Response to Reviewers.docx [file pone.0310650.s001.docx]

**Response to Reviewers**

Manuscript ID; PONE-D-23-34062

We sincerely appreciate your comments on our manuscript.

We have revised and improved our manuscript as suggested.

In the following, we provide a point-to-point response to every question raised. All answers are

represented in red.

**Reviewer#1**

Please ensure that your manuscript meets PLOS ONE's style requirements, including those for file naming. The PLOS ONE style templates can be found at 
https://journals.plos.org/plosone/s/file?id=wjVg/PLOSOne_formatting_sample_main_body.pdf and <https://journals.plos.org/plosone/s/file?id=ba62/PLOSOne_formatting_sample_title_authors_affiliations.pdf>. Response: We took the Plos one style template as a reference, double-spaced to my manuscript and numbered each line. And we have inserted six tables after the first paragraph of the relevant paper content.

**Reviewer#2**

In your Data Availability statement, you have not specified where the minimal data set underlying the results described in your manuscript can be found. PLOS defines a study's minimal data set as the underlying data used to reach the conclusions drawn in the manuscript and any additional data required to replicate the reported study findings in their entirety. All PLOS journals require that the minimal data set be made fully available. For more information about our data policy, please see http://journals.plos.org/plosone/s/data-availability.

""Upon re-submitting your revised manuscript, please upload your study’s minimal underlying data set as either Supporting Information files or to a stable, public repository and include the relevant URLs, DOIs, or accession numbers within your revised cover letter. For a list of acceptable repositories, please see http://journals.plos.org/plosone/s/data-availability#loc-recommended-repositories. Any potentially identifying patient information must be fully anonymized.

Important: If there are ethical or legal restrictions to sharing your data publicly, please explain these restrictions in detail. Please see our guidelines for more information on what we consider unacceptable restrictions to publicly sharing data: http://journals.plos.org/plosone/s/data-availability#loc-unacceptable-data-access-restrictions. Note that it is not acceptable for the authors to be the sole named individuals responsible for ensuring data access.

We will update your Data Availability statement to reflect the information you provide in your cover letter.

Response: The dataset, which is the minimal basis for the study, was uploaded as a public repositor by the Nagoya University Repository.

The uploaded data set is as follows.

Table 1　 <https://doi.org/10.18999/2008709>

Table 2	<https://doi.org/10.18999/0002008753>

Table 3	<https://doi.org/10.18999/2008754>

Table 4	<https://doi.org/10.18999/2008755>

Table 5	<https://doi.org/10.18999/2008756>

Table 6	<https://doi.org/10.18999/2008757>

**Reviewer#3**

PLOS requires an ORCID iD for the corresponding author in Editorial Manager on papers submitted after December 6th, 2016. Please ensure that you have an ORCID iD and that it is validated in Editorial Manager. To do this, go to ‘Update my Information’ (in the upper left-hand corner of the main menu), and click on the Fetch/Validate link next to the ORCID field. This will take you to the ORCID site and allow you to create a new iD or authenticate a pre-existing iD in Editorial Manager. Please see the following video for instructions on linking an ORCID iD to your Editorial Manager account:<https://www.youtube.com/watch?v=_xcclfuvtxQ>". Response: I got ORCID ID. The number is 0009-0002-0712-8798.

**Reviewer#4**

Your ethics statement should only appear in the Methods section of your manuscript. If your ethics

statement is written in any section besides the Methods, please move it to the Methods section and delete it from any other section. Please ensure that your ethics statement is included in your manuscript, as the ethics statement entered into the online submission form will not be published alongside your manuscript.

Response: We have confirmed that the statement of ethics is included in the methods of the

manuscript. The ethics statement entered in the online submission form was included in the

manuscript. We slightly corrected the sentence in red.

**Reviewer#4**

Please include your tables as part of your main manuscript and remove the individual files. Please note that supplementary tables (should remain/ be uploaded) as separate ""supporting information"" files". Response: We included the table as part of the manuscript and removed the individual files. We created the supplementary tables as separate "Supporting Information" files.

In addition, we reviewed the manuscript and revised the background and methodology to make the abstract more understandable. We Added missing information and fixed incorrect wording. The modifications are shown in red.

I checked the journal requirements and added or corrected them where necessary. Please let me know if there are any shortcomings or mistakes.

I am looking forward to hearing from you.

Kind regards,

Masako Sakamoto (PONE-D-23-34062)
