# Supplementary material for: Developing and validating a cross-cultural competence scale for Japanese nurses
Source: PLoS One. 2025 Jan 9;20(1):e0310650. doi: 10.1371/journal.pone.0310650 (PMC11717215; doi:10.1371/journal.pone.0310650)
Supplement: Attachment — Submitted filename: Response_to_Reviewer_2_.docx [file pone.0310650.s003.docx]

**Response to Reviewer 2**

**Manuscript ID: PONE-D-23-34062**

We sincerely appreciate your comments on our manuscript. We revised and improved our manuscript accordingly. Please see our point-to-point responses below. Our responses here and our revisions in the manuscript are represented in red.

**Question 1**

Page 9 - are these actual numbers of percentage?

Response: We revised as follows (line 46): “from 24.3% to 50.2%.”

**Question 2**

Page 12 - what is the ultimate purpose of developing and validating the Cross-Cultural Competence Scale for Japanese Nurses? This will highlight the usefulness of this study which can be added to the recommendations

Response: We clarified as follows (lines 93–100): “The ultimate purpose of developing and validating the Cross-cultural Competence Scale for Japanese Nurses (CCCSJN) was to create a tool that accurately visualizes the current state of cross-cultural competency among Japanese nurses. This will provide valuable data to guide improvements in cross-cultural competency, enhancing the ability of healthcare practitioners to effectively serve Japan’s growing and increasingly diverse foreign population, which has been expanding. As Japan continues to welcome more foreigners, cultural competence in healthcare is becoming increasingly essential, and this study helps to close that gap.

**Question 3**

add: "in caring for patients with different cultural backgrounds."
Response: We revised accordingly (lines 104–105): “To develop the CCCSJN, it was necessary to use questionnaire items that reflect the characteristics of Japanese nurses in caring for patients with different cultural backgrounds.”

**Question 4**

Page 13 - what were these three items that were removed?

Response:　The three excluded items were related to the utilization of social resources, confirmation, and dignity. We clarified as follows (lines 116–119): “The three items related to utilization of social resources, confirmation, and dignity were deleted because they were redundant with other questions or were difficult to understand, leaving 37 questions in the final draft.”

**Question 5**

Page 16 - How were these variables treated? You may include observations such as the group being homogenous (or not) and how this likely affected the results and implications on the generalizability of the findings.
Response: We treated the variables using the following steps, and descriptive statistics were used for variables related to the social attributes of the participants to understand their background. The median age of the participants was 33 years, and the median nursing experience was 9.8 years. The most common age of nurses in Japan was 40~49 years (mean = 41.3 years). The number of years of nursing experience ranged from 10 to 19 years (mean = 17.8 years). Regarding educational background, 83.7% had diplomas, and 11.2% held bachelor’s degrees. However, the educational background of nurses in Japan as a whole was 72.2% and 18.5%, respectively.

We revised as follows for additional clarity (lines 379–385): “Second, participants were about eight years younger and had eight years less nursing experience than nurses in Japan. Educational attainment was 11.5% higher for nurses with diplomas and 7.3% lower for nurses with bachelor’s degrees. In previous studies, age and nursing experience were significantly related to cross-cultural competence, and educational background may be related to cross-cultural understanding learning and foreign language proficiency. To confirm the accuracy of the CCCSJN, further studies with more-representative samples are needed.”

We also added four new references:

36. Japan Nursing Association. 2021 Fact-finding Survey of Nursing Staff: Japan Nursing Association Survey Research Report No. 98; 2022. https://www.[98.pdf (nurse.or.jp)](https://www.nurse.or.jp/nursing/home/publication/pdf/research/98.pdf)

37. Li J, He Z, Luo Y, Zhang R. Perceived transcultural self-efficacy of nurses in general hospitals in Guangzhou, China. Nurs Res. 2016;65: 371-379. https://www.doi.org/10.1097/NNR.0000000000000174

38. Tsui-Ting L, Miao-Yen C, Yu-Mei C, Mei-Hsiang L. A preliminary study on the cultural competence of nurse practitioners and its affecting factors. <http://www.dx.doi.org/10.3390/healthcare10040678>

39. Hietapakka L, Elovainio M, Wesolowska K, Aalto AM, Kaihlanen AM, Sinervo T, Heponiemi T. Testing the psychometric properties of the Finnish version of the cross-cultural competence instrument of healthcare professionals (CCCHP). BMC Health Serv Res. 2019;19: 294. https://www. doi.org.10.1186/s12913-019-4105-2

**Question 6**

You may also add here how cross-cultural variables were used in the study, esp in the analysis of findings.
Response: As suggested, we describe how the cross-cultural variables were used in this study. In this study, only descriptive statistics were used for cultural variables to understand participants’ background. Participants’ cross-cultural experience may affect their cross-cultural ability and can be used for comparative analysis with previous studies. In our next study, we plan to identify factors associated with intercultural competence by using cultural variables to discover factors associated with CCCSJN.

**Question 7**

Page 18 - This is the main study, right? If so, just edit to say, "In the main study" (this is to differentiate from the preliminary part where the initial items for the questionnaire were developed.

Response: We revised accordingly (line 212): “In the main study.”

**Question 8**

Page 27 - of variance on cultural competence.
Response: We revised as follows (lines 259–260): “The first to fifth factors contributed 32.53%, 6.54%, 5.33%, 3.69%, and 2.84% of the variance on cultural competence, respectively.”

**Question 9**

Page 29 -please compute again... this seems to be 86.5% response rate
Response: We revised accordingly (line 291): “86.5%”

**Question 10**

Page 29 - replace with "competence"
Response: We revised to “competence” (line 295).

**Question 11**

Why are the first two factors not discussed here? Cross-cultural understanding and communication ability are important factors and explain the highest variance in cultural competence of Japanese nurses. I suggest that these should also be mentioned in the discussion and how these are compared with other studies.
Response: As suggested, we revised as follows (lines 297–310): “The first factor is ‘cross-cultural understanding,’ which means understanding and accepting differences not only in cultures and customs but also in ways of thinking and values. Lack of understanding of cultural norms and traditions leads directly to inadequate information and poor-quality care [25]. Therefore, this scale indicates the importance of cross-cultural understanding to support patients’ cultural beliefs and values for Japanese nurses who are not yet confident in foreign nursing. In previous studies, factors related to cross-cultural understanding were also a major construct [6,18,27,28]. The second factor, ‘cross-cultural communication ability, comprises the communication skills of nurses so that foreign patients can receive healthcare without worry. Communication barriers reduce uptake and adherence to treatment and care and affect outcomes [26]. This factor included an easy-to-understand method to prevent misunderstandings due to language and cultural barriers and to check the degree of understanding. Communication ability was also a major component in previous studies [18,27,28,29].”

We also added the following references:

25. Shahzad S, Ali N, Younas A, Tayaben JL. Challenges and approaches to transcultural care: An integrative review of nurses’ and nursing students’ experiences. J Prof Nurs. 2021;37: 1119-1131. https://doi.org/10.1016/j.profnurs.2021.10.001

26. Gil S, Hooke MC, Niess D. The limited English proficiency patient family advocate role: Fostering respectful and effective care across language and culture in a pediatric oncology setting. J Pediatric Oncol Nurs. 2015;33: 190-198. https://www.doi.org/10.1177/1043454215611082

**Question 12**

Page 33
You may also look into the socio-demographic characteristics and cross-cultural experiences of the sample in the study for sources of study limitations.　　　　　　　　　　　　　　　　　　　　　　　　Response: We revised as follows for additional clarity (lines 370–376): “The number of foreign residents in Aichi Prefecture, where the current participants work, was second only to Tokyo; therefore, more people had experience in providing care for foreign patients than in other prefectures. Nurses working in areas with few foreign residents are expected to have less experience providing care for foreign patients. Since cross-cultural competence was significantly related to the experience of caring for foreign patients, the influence of locality on cross-cultural competence should also be carefully observed.”

In addition to the above, the following revisions were made:

- Revised “cross-cultural communication skill” and “collaboration with multiple professions” to “cross-cultural communication ability” and “cooperation with multiple professions” on lines 32–33, 264–265, and 283–285.
- Revised “51.95” and “0.92” to “50.92” and “0.94” on line 34-35.
- Added “potential” to line 134.
- Revised “≥” to “>” on line 193.
- Revised “Pearson correlation coefficients” to “Spearman rank correlation coefficients” on line 198.
- Revised part of Table 2 on lines 229–230.
- Revised to “0.63–0.85”, “0.41–0.76”, “0.41–0.82”, “0.55–0.81” and 0.42–0.93 to “0.62–0.84”, “0.40–0.71”, ”0.44–0.71”, “0.55–0.79” and “0.42–0.92” on line 261-263.
- Revised “r” to “rs” on line 276.
- Revised “r = 0.61” to “rs = 0.69” on line 278.
- Revised part of Table 6 on lines 279–280.
- Revised “0.92”, “0.91”, “0.75”, “0.82” and 0.79 to “0.94”, “0.94”, “0.82”, “0.88” and “0.84” on line 282 -285.
- Revised “ability” to “competence” on line 295.
- Revised “collaboration” to “cooperation” on lines 322 and 330.
- Revised “use” to “utilization” on line 340.
- Revised “0.92” and “0.75–0.91” to “0.94” and “0.78-0.94” on line 357-358.

We carefully reviewed your comments and made key revisions when necessary. Please let us know if there are any further concerns. We look forward to hearing from you.

Kind regards,

Masako Sakamoto (PONE-D-23-34062)
